# Frontotemporal lobar degeneration targets brain regions linked to expression of recently evolved genes

**DOI:** 10.1093/brain/awae205

**Published:** 2024-06-28

**Authors:** Lorenzo Pasquini, Felipe L Pereira, Sahba Seddighi, Yi Zeng, Yongbin Wei, Ignacio Illán-Gala, Sarat C Vatsavayai, Adit Friedberg, Alex J Lee, Jesse A Brown, Salvatore Spina, Lea T Grinberg, Daniel W Sirkis, Luke W Bonham, Jennifer S Yokoyama, Adam L Boxer, Joel H Kramer, Howard J Rosen, Jack Humphrey, Aaron D Gitler, Bruce L Miller, Katherine S Pollard, Michael E Ward, William W Seeley

**Affiliations:** Department of Neurology, Memory and Aging Center, University of California, San Francisco, CA 94158, USA; Department of Neurology, Neuroscape, University of California, San Francisco, CA 94158, USA; Department of Neurology, Memory and Aging Center, University of California, San Francisco, CA 94158, USA; National Institute of Neurological Disorders and Stroke, Neurogenetics Branch, Bethesda, MD 20892, USA; Department of Genetics, Stanford University School of Medicine, Stanford, CA 94305, USA; School of Artificial Intelligence, Beijing University of Posts and Telecommunications, Beijing 100876, China; Department of Neurology, Memory and Aging Center, University of California, San Francisco, CA 94158, USA; Global Brain Health Institute, University of California, San Francisco, San Francisco, CA, 94158USA; Trinity College Dublin, Dublin D02 X9W9, Ireland; Department of Neurology, Hospital de la Santa Creu i Sant Pau, Biomedical Research Institute, Universitat Autònoma de Barcelona, Barcelona, Catalunya, 08041, Spain; Department of Neurology, Memory and Aging Center, University of California, San Francisco, CA 94158, USA; Department of Neurology, Memory and Aging Center, University of California, San Francisco, CA 94158, USA; Global Brain Health Institute, University of California, San Francisco, San Francisco, CA, 94158USA; Trinity College Dublin, Dublin D02 X9W9, Ireland; Department of Neurology, Memory and Aging Center, University of California, San Francisco, CA 94158, USA; Department of Neurology, Memory and Aging Center, University of California, San Francisco, CA 94158, USA; Department of Neurology, Memory and Aging Center, University of California, San Francisco, CA 94158, USA; Department of Neurology, Memory and Aging Center, University of California, San Francisco, CA 94158, USA; Department of Pathology, University of California, San Francisco, CA 94158, USA; Department of Neurology, Memory and Aging Center, University of California, San Francisco, CA 94158, USA; Department of Neurology, Memory and Aging Center, University of California, San Francisco, CA 94158, USA; Department of Radiology, University of California, San Francisco, CA 94158, USA; Department of Neurology, Memory and Aging Center, University of California, San Francisco, CA 94158, USA; Department of Radiology, University of California, San Francisco, CA 94158, USA; Department of Neurology, Memory and Aging Center, University of California, San Francisco, CA 94158, USA; Department of Neurology, Memory and Aging Center, University of California, San Francisco, CA 94158, USA; Department of Neurology, Memory and Aging Center, University of California, San Francisco, CA 94158, USA; Nash Family Department of Neuroscience and Friedman Brain Institute, Icahn School of Medicine at Mount Sinai, New York, NY 10029, USA; Department of Genetics, Stanford University School of Medicine, Stanford, CA 94305, USA; Department of Neurology, Memory and Aging Center, University of California, San Francisco, CA 94158, USA; Gladstone Institute of Data Science and Biotechnology, San Francisco, CA 94158, USA; Institute for Human Genetics, University of California San Francisco, San Francisco, CA 94158, USA; Department of Epidemiology and Biostatistics and Bakar Institute for Computational Health Sciences, University of California San Francisco, San Francisco, CA 94158, USA; Chan Zuckerberg Biohub, San Francisco, CA 94158, USA; National Institute of Neurological Disorders and Stroke, Neurogenetics Branch, Bethesda, MD 20892, USA; Department of Neurology, Memory and Aging Center, University of California, San Francisco, CA 94158, USA; Department of Pathology, University of California, San Francisco, CA 94158, USA

**Keywords:** frontotemporal lobar degeneration, cryptic exon, human accelerated regions, TDP-43, tau, gene expression

## Abstract

In frontotemporal lobar degeneration (FTLD), pathological protein aggregation in specific brain regions is associated with declines in human-specialized social-emotional and language functions. In most patients, disease protein aggregates contain either TDP-43 (FTLD-TDP) or tau (FTLD-tau). Here, we explored whether FTLD-associated regional degeneration patterns relate to regional gene expression of human accelerated regions (HARs), conserved sequences that have undergone positive selection during recent human evolution.

To this end, we used structural neuroimaging from patients with FTLD and human brain regional transcriptomic data from controls to identify genes expressed in FTLD-targeted brain regions. We then integrated primate comparative genomic data to test our hypothesis that FTLD targets brain regions linked to expression levels of recently evolved genes. In addition, we asked whether genes whose expression correlates with FTLD atrophy are enriched for genes that undergo cryptic splicing when TDP-43 function is impaired.

We found that FTLD-TDP and FTLD-tau subtypes target brain regions with overlapping and distinct gene expression correlates, highlighting many genes linked to neuromodulatory functions. FTLD atrophy-correlated genes were strongly enriched for HARs. Atrophy-correlated genes in FTLD-TDP showed greater overlap with TDP-43 cryptic splicing genes and genes with more numerous TDP-43 binding sites compared with atrophy-correlated genes in FTLD-tau. Cryptic splicing genes were enriched for HAR genes, and vice versa, but this effect was due to the confounding influence of gene length. Analyses performed at the individual-patient level revealed that the expression of HAR genes and cryptically spliced genes within putative regions of disease onset differed across FTLD-TDP subtypes.

Overall, our findings suggest that FTLD targets brain regions that have undergone recent evolutionary specialization and provide intriguing potential leads regarding the transcriptomic basis for selective vulnerability in distinct FTLD molecular-anatomical subtypes.

## Teaser

Frontotemporal lobar degeneration (FTLD) erodes brain functions that have evolved relatively recently in humans, such as language and social behaviour. Pasquini *et al*. explore whether brain regions targeted by FTLD are linked to expression of genes that have undergone positive selection during human evolution.

**See Shen *et al*. (https://doi.org/10.1093/brain/awae267) for a scientific commentary on this article.**

## Introduction

Frontotemporal dementia (FTD) refers to a group of clinical syndromes linked to underlying frontotemporal lobar degeneration (FTLD) pathology. These FTD syndromes present with deficits in recently evolved social-emotional and language functions,^1,2^ resulting from degeneration within networks anchored by frontal, insular and anterior temporal brain regions.^3,4^ The most common FTD syndromes are the behavioural variant (bvFTD) characterized by social-emotional deficits^2^; semantic variant primary progressive aphasia (svPPA), which presents with loss of knowledge related to words, objects and emotions^1,5^; non-fluent variant PPA (nfvPPA), characterized by speech production difficulties^1,5^; corticobasal syndrome, an asymmetric akinetic-rigid syndrome associated with a progressive loss of limb controls^4^; and progressive supranuclear palsy-Richardson syndrome (PSP-RS), associated with oculomotor deficits and gait instability.^4^ FTLD pathology, in turn, can be divided into three major molecular classes based on whether the neuronal and glial inclusions are composed of tau (FTLD-tau), TDP-43 (FTLD-TDP) or FUS/EWS/TAF15 (FET) family proteins.^6^ Each major molecular class can be further divided into distinct subtypes based on the pathomorphology and distribution of the inclusions across regions, layers and cell types. Factors driving the selective predilection of each FTLD subtype for distinct, yet overlapping, anatomical structures remain unknown.

Comparative genomic studies have the potential to shed light on the relationship between brain evolution and the erosion of human-specialized functions in FTLD. Novel genome-wide studies have identified a unique set of loci that are conserved across mammalian evolution, indicating important biological roles, but significantly diverged in the human lineage, suggesting changes to those roles in humans compared to chimpanzees and other primates.^7,8^ These short sequences, referred to as human accelerated regions (HARs), are commonly located in non-coding DNA, often near genes associated with transcription and DNA binding.^9^ Several studies have linked HARs to the emergence of distinctive human traits, such as the opposable thumb,^8^ language and social behaviour,^10^ and mutations in HAR genes are observed in neuropsychiatric^11,12^ and neurodevelopmental disorders.^7^

Because FTLD is pathologically heterogeneous, it is possible that FTLD-TDP and FTLD-tau intersect with brain evolution through distinct, yet overlapping mechanisms. The role of protein aggregation has been a major focus of research, but in FTLD-TDP, TDP-43 aggregation is almost always accompanied, and may be preceded, by loss of nuclear TDP-43 expression.^13-16^ TDP-43, encoded by *TARDBP*, is an RNA-binding protein predominantly expressed in the nucleus of healthy neurons,^17-19^ where it plays diverse roles in transcription regulation by binding to RNAs rich in GU repeats. One role is to suppress incorporation of cryptic exons, short stretches of non-conserved intronic RNA that are normally removed during pre-mRNA splicing.^20-23^ Upon TDP-43 loss-of-function, cryptic exons are spliced into messenger RNAs, often introducing premature stop codons or frame shifts that result in nonsense mediated decay and loss of normal protein expression.^24-26^ To date, >1000 potential genes subject to cryptic splicing have been identified, yet, despite some compelling candidates,^17,24^ it remains unknown which cryptic splicing events, if any, are most relevant to FTLD-TDP pathogenesis. Crucially, although TDP-43 is a highly conserved protein, cryptic splicing genes show almost no overlap when comparing mice to humans,^20^ suggesting that TDP-43 may have adopted a unique regulatory role across the human evolutionary lineage.

Here, we combined neuroimaging, neuropathological, average regional brain transcriptomic, comparative genomic and cell biological data to identify genes whose expression pattern, in the healthy brain, correlates with cortical atrophy patterns in FTLD and to test the hypothesis that HAR genes are expressed in patterns that correlate with FTLD atrophy. We further explored associations between expression patterns for TDP-43 cryptic splicing genes and FTLD-associated atrophy and the overlap between HAR and cryptic splicing genes.

## Materials and methods

### Participants

We searched the University of California, San Francisco (UCSF) Neurodegenerative Disease Brain Bank database for participants autopsied between 2008 and 2020 who had at least one high quality structural MRI scan prior to death; this search yielded 322 participants. From this pool, we included patients with a primary neuropathological diagnosis of FTLD-TDP (types A–C) or FTLD-tau [corticobasal degeneration (CBD) or Pick's disease]. Although PSP is a common subtype of FTLD-tau, we omitted it here because it is characterized by a predominantly subcortical atrophy pattern not well suited to our transcriptomic data (see later). Patients were excluded if they had: (i) Braak neurofibrillary tangle stage^27^ >3; (ii) Alzheimer's disease neuropathological change^28^ >intermediate; (iii) Lewy body disease^29^ >brainstem predominant; or (iv) major territorial ischaemic infarcts or intracranial haemorrhages.^30^ Based on these stringent criteria, 28 patients with TDP-A, 35 with TDP-B, 29 with TDP-C, 45 with CBD and 27 with Pick's disease were included in the study. In accordance with the Declaration of Helsinki, patients or their surrogates provided written informed consent prior to participation, including consent for brain donation. The UCSF Committee on Human Research approved the study.

All patients received clinical diagnoses by a multidisciplinary team following thorough neurological, neuroimaging and neuropsychological assessments. Clinical severity was assessed using the Mini-Mental State Examination^31^ and the Clinical Dementia Rating scale total and sum of boxes scores, using a version of the Clinical Dementia Rating scale adapted for FTD.^32^ Patient demographics, as well as clinical and neuropathological data, are provided in Table 1. Race and ethnicity, handedness and years of education were self-reported by patients or surrogates.

**Table 1:** Pathological, clinical and demographic characteristics of patients with FTLD

Pathological diagnoses	TDP-A	TDP-B	TDP-C	CBD	Pick's disease
*n*	28	35	29	45	27
Age at MRI scan in years	63.1 (6.7)	62.1 (9.1)	64.3 (7.4)	64.5 (6.7)	64.4 (7.1)
Scan-death interval in years	4.7 (3.2)	2.6 (2.2)	7.4 (2.8)	3.2 (1.6)	5.1 (3.4)
Sex (female:male)	16:12	15:20	11:18	25:20	11:16
Handedness (ambidextrous:left:right)	0:2:26	0:2:33	0:3:26	0:0:45	0:4:23
Education in years	16.1 (3.4)	16.1 (3.0)	16.6 (3.0)	16.2 (2.9)	16.0 (2.7)
Race (Asian:Black:White:n.a.)	1:0:24:3	0:0:34:1	0:0:28:1	4:1:36:4	2:0:22:3
Ethnicity (Hispanic/Latino:n.a.)	0:13	2:12	1:3	1:16	2:6
Scanner type (1.5 T:Trio:Prisma:4 T)	13:12:0:3	9:16:3:7	21:5:0:3	16:24:1:4	14:10:0:3
Total intracranial volume (l)	1.4 (0.1)	1.4 (0.1)	1.5 (0.2)	1.5 (0.1)	1.5 (0.2)
Clinical Dementia Rating (CDR)	1.1 (0.8)	1.4 (0.8)	0.7 (0.6)	0.8 (0.6)	0.8 (0.6)
CDR Sum of Boxes	6.1 (4.3)	7.4 (4.2)	3.8 (3.0)	4.1 (3.6)	5.3 (3.4)
MMSE	22.4 (7.2)	24.2 (5.4)	23.3 (7.2)	24.3 (5.8)	22.8 (8.0)
**Clinical diagnoses**					
Alzheimer's disease-type dementia	0	1	0	2	1
Behavioural variant FTD	16	30	2	11	16
Corticobasal syndrome	3	0	0	15	2
Mild cognitive impairment	0	1	1	0	0
Non-fluent variant PPA	4	1	0	11	6
Posterior supranuclear palsy syndrome	0	0	0	6	0
Semantic variant PPA	5	2	26	0	2

CBD = corticobasal degeneration; FTD = frontotemporal dementia; FTLD = frontotemporal lobar degeneration; MMSE = Mini-Mental State Examination; n.a. = not available; PPA = primary progressive aphasia.

### Neuropathology

Post-mortem research-oriented autopsies were performed at the UCSF Neurodegenerative Disease Brain Bank. Neuropathological diagnoses were made following consensus diagnostic criteria^6,29,33^ based on standard histological and immunohistochemical methods.^34,35^

### Structural neuroimaging

#### Acquisition and preprocessing

Because patients were evaluated over an 18-year interval, MRI scans were obtained on different scanners over time. For 71 patients, T_1_-weighted magnetization prepared rapid gradient echo (MPRAGE) MRI sequences were acquired at the UCSF Neuroscience Imaging Center, either on a 3 T Siemens Tim Trio (*n* = 67) or a 3 T Siemens Prisma Fit scanner (*n* = 4). Both scanners had similar acquisition parameters (sagittal slice orientation; slice thickness = 1.0 mm; slices per slab = 160; in-plane resolution = 1.0 × 1.0 mm; matrix = 240 × 256; repetition time = 2300 ms; inversion time = 900 ms; flip angle = 9°), although echo time differed slightly (Trio: 2.98 ms; Prisma: 2.9 ms). For the remaining 93 patients, MRI scans were obtained at the San Francisco Veterans Affairs Medical Center using MPRAGE sequences acquired either on a Siemens 1.5 T Magnetom scanner (*n* = 73; voxel resolution 1.0 × 1.0 × 1.5 mm; repetition time = 10 ms; echo time = 4 ms; inversion time = 300 ms; flip angle = 15) or on a Siemens 4 T Magnetom scanner (*n* = 20; voxel resolution 1.0 × 1.0 × 1.0 mm; repetition time = 2.3 ms; echo time = 3 ms; inversion time = 950 ms; flip angle = 7). The average interval between scanning date and autopsy was 4.8 ± 2.6 years.

All structural magnetic resonance images underwent a voxel-based morphometry analysis^36^ after being visually inspected for motion and scanning artefacts. Structural images were segmented in grey matter, white matter and CSF, and normalized to MNI space using SPM12 (http://www.fil.ion.ucl.ac.uk/spm/software/spm12/). Grey matter images were modulated by dividing the tissue probability values by the Jacobian of the warp field. The resulting images of voxel resolution 2.0 × 2.0 × 2.0 mm were smoothed with an isotropic Gaussian kernel with a full-width at half-maximum of 8 mm.

#### W-score maps

To generate participant-specific atrophy maps, the smoothed grey matter images were transformed to W-score maps. W-score maps are voxel-wise statistical maps that reflect levels of atrophy for each individual after adjustment for relevant covariates.^37,38^ This approach is particularly useful when performing analyses with heterogenous samples as grey matter atrophy estimates are adjusted for demographical and methodological nuisance covariates, including age, gender, scanner type and total intracranial volume.^38^ The W-score model used for this study was published^39^; it involved 397 healthy older controls (Supplementary Table 1) and included age at MRI, sex, years of education, handedness, scanner type and total intracranial volume as covariates. Briefly, multiple regression analyses at the voxel-wise level were performed on the normative dataset to estimate grey matter intensity as adjusted for age, sex, years of education, handedness, MRI scanner and total intracranial volume. This model was then used to estimate the expected grey matter intensity for a patient by using their demographical data. The discrepancy between the raw and the expected maps is used to derive the W-score map:


$$W=\frac{\left(Raw-Expected\right)}{SD\varepsilon }$$

where *Raw* is the raw value of a voxel from the smoothed image of a patient; *Expected* is the expected value for the voxel of that specific patient based on the healthy control model; and *SDε* is the standard deviation of the residuals from the healthy control model. Additional analyses were performed to assess whether the W-score model effectively mitigated the impact of scanner type in the generation of W-score maps (Supplementary Fig. 1, Supplementary Table 2 and Supplementary material, ‘Findings’ file).

### Gene expression

#### Allen human brain atlas

Average human brain gene expression data were derived from microarray data available through the Allen Human Brain Atlas (AHBA) (http://human.brain-map.org/static/download). As described in detail elsewhere,^40^ tissue samples were extracted across both hemispheres from two human brain donors, as well as the left hemisphere of four additional donors, totalling 3702 tissue samples. Microarray analysis quantified gene expression across 58 692 probes, providing an estimate of the relative expression of 20 734 genes within the tissue samples.^41^ The publicly available toolbox abagen (https://github.com/rmarkello/abagen)^42^ was then used for: (i) updating probe-to-gene annotations using the latest available data; (ii) data filtering, where expression values that do not exceed background are removed; (iii) probe selection, which, for genes indexed by multiple probes, involves selecting a single representative measure to represent the expression of that gene across all donor brains; and (iv) sample assignment, where tissue samples from the AHBA were mapped to 273 parcels of the Brainnetome brain regional parcellation atlas (https://atlas.brainnetome.org/).^43^ Expression values of regions in the left hemisphere were not mirrored to homologous regions in the right hemisphere, due to the predilection of distinct FTLD subtypes to target one hemisphere versus the other (e.g. FTLD-TDP-C for the left hemisphere). See the online Supplementary material ‘Appendix’ section for a more detailed report on the procedures using abagen. This atlas was selected because it was derived using functional and structural anatomy and connectivity, and it includes paired homologous bilateral regions. Steps (i–iv) were followed by (v) normalization of expression measures to account for inter-individual differences and outlying values; (vi) gene-set filtering, to remove genes that are inconsistently expressed across the six brains; and (vii) averaging gene expression across donors. This procedure resulted in 15 655 genes (Supplementary material) and their average expression in 273 brain parcels. Finally, we removed subcortical regions (cerebellum, thalamus, basal ganglia and hippocampus) due to substantial differences in their gene expression values when compared to cortical parcels,^44^ yielding a 202 regions × 15 655 genes matrix (Supplementary Fig. 2 and Supplementary material, ‘Data’ section).

#### Human accelerated region genes

HARs were taken from a comparative genomic analysis that identified, from a list of several recent publications, loci with accelerated divergence in humans when compared to chimpanzees.^7^ A total of 2737 HARs were identified, representing 2164 unique HAR-associated genes.^7^ Of these 2164 HAR genes, 1373 were identified as sufficiently expressed in the brain based on the AHBA brain-expressed gene dataset (probes used if they exceeded background signal for >50% of all samples) and used in our analyses, referred to simply as HAR genes^45^ (Supplementary material, ‘Data’ section).

#### Cryptic splicing genes

Three prior studies contributed to the list of TDP-43-dependent cryptic splicing genes^24-26^. These studies were chosen because they identified cryptic splicing targets either based on FTLD-TDP human post-mortem tissue data^24^ or induced pluripotent stem cell (iPSC)-derived neuronal cell lines,^25,26^ omitting studies based on non-neuronal cells. For the tissue-based study, splicing analyses were performed on RNA sequencing data from TDP-43-positive and TDP-43-negative neuronal nuclei isolated from frontal cortices of seven patients with FTLD-TDP. This approach identified 66 cryptic splicing genes,^24^ of which 63 genes exceeded the brain expression threshold imposed on the AHBA data. In a separate study, RNA sequencing was performed on human iPSC-derived cortical-like neurons, in which TDP-43 expression was reduced using clustered regularly interspaced short palindromic repeats interference (CRISPRi).^25^ Differential splicing and expression analyses based on this dataset followed by splice junction evaluation via visual inspection of sashimi plots revealed 107 genes with putative cryptic splicing events, of which 97 also exceeded the brain expression threshold in the AHBA dataset. Finally, a third study performed a comprehensive analysis of differential splicing events in TDP-43 depleted versus control iPSC-derived neurons to develop a high-quality neuronal cryptic exon atlas.^26^ This analysis revealed 233 cryptic splicing genes, of which 201 exceeded the brain expression threshold in the AHBA brain-expressed gene dataset, as previously defined. By combining the two iPSC-derived neuronal cryptic splicing gene sets, 216 unique cryptic splicing genes were identified and combined with the post-mortem tissue-derived cryptic splicing genes to yield a final brain-expressed cryptic splicing gene set of 257 unique genes (Supplementary material, ‘Data’ section).

In addition, we identified brain-expressed genes containing higher amounts of GU repeats, which serve as putative regulatory binding sites for TDP-43. The *Bioconductor BiomaRt* package in R was used to retrieve the genetic sequences of brain-expressed genes, which were available for 15 244 of 15 655 brain-expressed genes. The presence of GU repeats (GU tetramers, pentamers and hexamers) in these brain-expressed genes was estimated using the *universalmotiv* function in R, which queried for GT repeats in the DNA sequences. As GU repeats were found in a majority of brain-expressed genes (14 117/15 244 or 93% of genes), we increased the likelihood that genes are regulated by TDP-43 by identifying those GU repeat-containing genes that contain higher amounts of putative TDP-43 binding sites. This was achieved by identifying a set of 7792 genes that contain higher amounts of GU repeats, defined as above-median content of GU tetramers, pentamers or hexamers.

#### Spatial correlation analyses

We derived group-averaged, voxel-wise W-score maps for each FTLD pathological subtype (TDP-A, TDP-B, TDP-C, CBD and Pick's disease). For each subtype-averaged map, we then averaged the W-score values of voxels contained in each cortical Brainnetome parcel to derive a vector reflecting the level of regional cortical atrophy in each FTLD subtype. These FTLD subtype-specific atrophy vectors were then separately correlated with the cortical gene expression levels derived from the AHBA brain-expressed gene dataset using Pearson's correlation coefficients. This procedure enabled us to assess the spatial similarity between each FTLD subtype atrophy pattern and the cortical expression levels of 15 655 brain-expressed genes from the AHBA.^44,46,47^ Similar analyses were performed with a publicly available W-score map of cortical atrophy from 147 amyloid- and tau-confirmed patients with Alzheimer's disease-type dementia^48^ (Supplementary material, ‘Data’ section and Supplementary Fig. 7).

Because spatial autocorrelation inherent to neuroimaging data can inflate *P*-values in brain map analyses,^49^ we corrected for this autocorrelation by applying a bootstrap-based approach (Supplementary Fig. 3), in which 5000 surrogate maps that preserve the autocorrelation properties of the FTLD atrophy maps were generated using the toolbox BrainSMASH (https://brainsmash.readthedocs.io/en/latest/approach.html). We then derived autocorrelation-corrected *P*-values by counting how often the correlation between surrogate atrophy maps and each gene map was equal to or higher than the true correlation and divided this number by the total number of bootstraps. Only false discovery rate (FDR) adjusted *P*-values < 0.05 were considered significant. For control analyses, we also generated lists with a more stringent threshold (FDR adjusted *P* < 0.01; Supplementary material, ‘Data’ section and Supplementary Fig. 6). This procedure enabled us to identify genes with spatial expression patterns resembling the FTLD atrophy maps.

To remove genes displaying spurious correlations to FTLD-TDP atrophy maps from subsequent analyses, we assessed how applying a threshold based on the absolute correlation value would affect the list of selected genes significantly correlating with FTLD-TDP atrophy maps. We applied absolute correlation thresholds between 0 and 0.5 in steps of 0.05 and derived an index assessing how each threshold impacted the ratio between the number of genes uniquely associated with each FTLD-TDP atrophy map and the number of genes that significantly correlated with at least two FTLD-TDP subtype atrophy maps:


$$\begin{array}{rl} & Uniqueness_{index}=\\ & \frac{\left(Unique_{TDP-A}+Unique_{TDP-B}+Unique_{TDP-C}\right)}{\left(Shared_{TDP-AB}+Shared_{TDP-BC}+Shared_{TDP-AC}+Shared_{TDP-ABC}\right)} \end{array}$$

This uniqueness index was used to identify 0.2 as the optimal absolute correlation threshold to better identify subtype-specific correlating genes while at the same time preserving longer gene lists. Genes displaying correlation values between 0.2 and −0.2 were hence considered as not being sufficiently correlated with FTLD-TDP atrophy maps and discarded from further analyses regardless of the associated *P*-value. We then assessed the overlap between gene lists representing FTLD atrophy-correlated, HAR and cryptic splicing genes through one-tailed Fisher's exact tests using the *fisher.test* function in R. The 15 565 brain-expressed genes from the AHBA dataset were used as background genes, unless specified otherwise.

#### Gene set enrichment analysis

Gene set enrichment analyses are computational methods that query large genomic databases to determine whether an *a priori* defined set of genes shows statistically significant associations with known biological pathways. We ran five separate gene set enrichment analyses^50^ leveraging the correlation values (FDR adjusted *P* < 0.05) between brain genes and atrophy maps of FTLD-TDP and FTLD-tau subtypes (one analysis for each subtype). We performed gene set enrichment analyses using the *clusterProfiler* package in R software v4.2.1 for Gene Ontology^51^ and for Kyoto Encyclopedia of Genes and Genomes (KEGG).^52^ We used the 15 655 brain-expressed genes from AHBA as the background list. We then focused on terms and pathways with a Holm adjusted *P*-value of <0.05 shared by a majority of FTLD subtypes. Correlation matrices were used to reflect the strength of correlation between genes associated with various KEGG terms and FTLD-TDP and FTLD-tau subtype atrophy patterns (FDR adjusted *P* < 0.05).

#### Graph network

Over the course of the study, we identified a set of genes that correlated with FTLD-TDP atrophy and overlapped with the previously introduced cryptic splicing (CS) and HAR gene lists. Graph theoretical approaches were applied to this list of 23 CS-HAR genes correlating with FTLD-TDP atrophy to generate topographical representations of gene expression networks.^53^ The regional expression values of selected genes were correlated with each other to generate a gene-to-gene regional co-expression matrix. This matrix was binarized at a Pearson's correlation coefficient threshold of 0.3 and the number of surviving edges between genes was counted for each gene of interest to derive a measure of nodal degree, reflecting the level of connectedness between a specific gene and each other gene selected for the analysis. Resulting data were rendered as a graph.

#### Gene length

Standard *BiomatRt* code in R (https://useast.ensembl.org/info/data/biomart/index.html) leveraging the widely used Ensembl genome browser (https://ensemblgenomes.org/) was used to derive the full coordinates of exonic and intronic sequences of brain-expressed genes used in our analyses (BSgenome.Hsapiens.UCSC.hg38). This information was used to estimate the length of HAR genes used in our analyses and to identify a set of 1353 length-matched non-HAR genes through nearest neighbour matching using the *matchit* library in R.

#### Disease epicentres

We used an approach previously applied by our group and others^54-57^ to identify patient-tailored epicentres. This method defines an epicentre as the brain region whose normative functional (task-free) connectivity map most closely resembles the patient's atrophy W-map. Normative connectivity maps were derived using task-free functional MRI data from a cohort of 75 healthy older subjects to generate a library of 194 intrinsic functional connectivity maps from seed regions of the Brainnetome atlas spanning the entire cerebral cortex. Characterization of the normative sample and methodological details are presented elsewhere.^54^ We then compared each patient's atrophy W-map with this library to select the seed that generated a functional connectivity map showing the highest spatial (Pearson) correlation to the subject-specific atrophy W-map. Subtype-specific frequency maps were used to quantify the spatial distribution of epicentres in FTLD-TDP, as group-level atrophy in FTLD-TDP was associated with both cryptic splicing and HAR genes. Finally, we used Wilcoxon signed-rank tests to assess whether the expression of cryptic splicing and HAR genes within disease epicentres differed across FTLD-TDP subtypes. For each gene, epicentres’ gene expression levels were then averaged for each subtype to derive a cross-fold change score reflecting higher or lower gene expression in one group versus the other.

## Results

### FTLD subtypes target brain regions expressing shared and distinct genes

**Figure 1:**
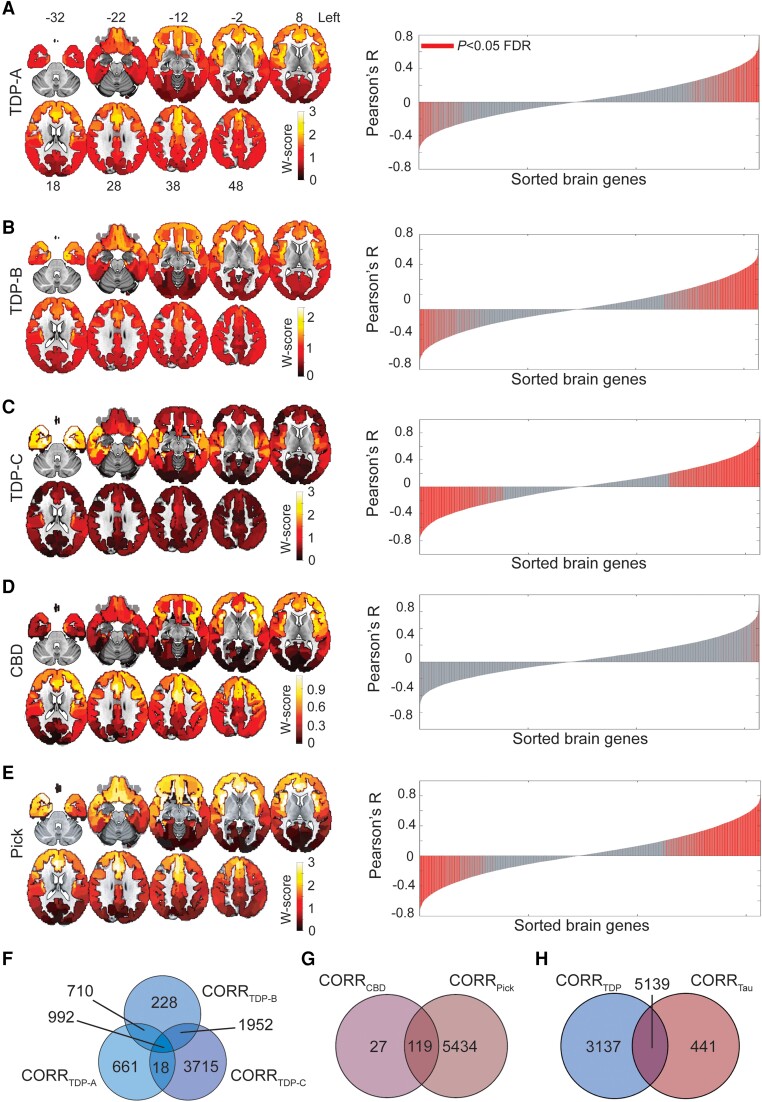
**Regional gene expression correlating with atrophy in FTLD subtypes**. (**A**–**E**) Maps on the *left* show group-averaged W-score maps for each frontotemporal lobar degeneration (FTLD) subtype; warmer colours reflect greater cortical grey matter atrophy. Bar plots on the *right* show the Pearson correlation coefficients between the regional gene expression and regional atrophy in FTLD subtypes; red bars indicate significantly correlated genes, False Discovery Rate (FDR) adjusted *P* < 0.05. The left hemisphere is shown on the *right*. (**F**) Several genes were shared among FTLD-TDP subtypes, although a conspicuous number of genes was uniquely correlated to each subtype, particularly to FTLD-TDP-C. (**G**) While FTLD-Pick showed many uniquely correlated genes, most genes correlating with atrophy in FTLD-CBD were shared with FTLD-Pick. (**H**) FTLD-TDP had many uniquely correlated genes, while a large proportion of genes correlating with atrophy in FTLD-tau were shared with FTLD-TDP. CORR_FTLD-subtype_ indicates lists of genes correlating with that specific FTLD subtype. CBD = corticobasal degeneration.

We first sought to explore the relationship between FTLD atrophy topography and regional gene expression in the healthy brain. We included the three major FTLD-TDP subtypes (A–C) and the two major sporadic FTLD-tau subtypes, CBD and Pick's disease, with predominant cortical involvement. First, we replicated the atrophy patterns associated with these disorders using *in vivo* brain MRI data from patients with an autopsy-based diagnosis.^58,59^ Structural MRI volumes were segmented into grey matter tissue probability maps and further transformed to W-score maps,^37,38^ where higher W-scores reflect more severe voxel-wise atrophy for each patient, adjusted for demographic variables. FTLD subtype-specific cortical atrophy maps were derived by averaging W-score maps within each group (Fig. 1A–E, left), revealing a more dorsal frontal and insular pattern in TDP-A; a more ventral pattern in TDP-B, with less severe atrophy overall; an anterior temporal-predominant pattern in TDP-C; a milder, predominantly frontal atrophy pattern in CBD; and a severe frontal and insular pattern of atrophy in Picks’ disease.

We next leveraged gene expression microarray data from the AHBA to identify genes whose cortical expression pattern resembles cortical grey matter atrophy characteristic of each FTLD subtype. Starting with a matrix of 15 655 genes and their regional expression in 202 cortical brain parcels (Supplementary Fig. 2), we performed spatial correlation analyses to associate group-level grey matter atrophy patterns with average regional expression levels of brain-expressed genes. We used a bootstrap-based test, correcting for the spatial autocorrelations inherent to neuroimaging data,^49^ to delineate genes significantly correlated with atrophy in each FTLD subtype (FDR adjusted *P* < 0.05, Fig. 1A–E, red bars in the right panels). To remove spurious correlations from further analyses, we only considered absolute correlation values ≥ 0.2, based on a uniqueness index maximizing the number of genes uniquely associated with each FTLD subtype (Supplementary Fig. 3). This procedure identified unique and shared genes correlating with atrophy across FTLD subtypes. TDP-C showed the highest number of uniquely correlated genes (3715) among the FTLD-TDP subtypes, followed by TDP-A (661 genes) and TDP-B (228 genes). A total of 992 genes were shared between all FTLD-TDP subtypes (Fig. 1F). Among FTLD-tau, CBD showed only 27 uniquely correlated genes, due to the mild frontal atrophy found in this sample highly resembling the inherent frontal-posterior spatial autocorrelation gradient inherent to neuroimaging data.^49^ Pick's disease showed the most uniquely correlated genes (5434) among all FTLD subtypes. CBD shared 119 genes with Pick's disease, which yielded a total of 5434 uniquely correlated genes for FTLD-tau (Fig. 1G). When pooling both FTLD-TDP and FTLD-tau together, 3137 genes were uniquely correlated with at least one TDP subtype, 441 with at least one tau subtype, and 5139 were shared among TDP-43 and tau FTLD subtypes (Fig. 1H). While positively correlated genes showed high expression levels in atrophied brain regions and low expression levels in spared areas, negatively correlated genes showed the opposite patter, showing high expression levels in spared brain regions and low expression levels in atrophied areas (Supplementary Fig. 4).

**Figure 2:**
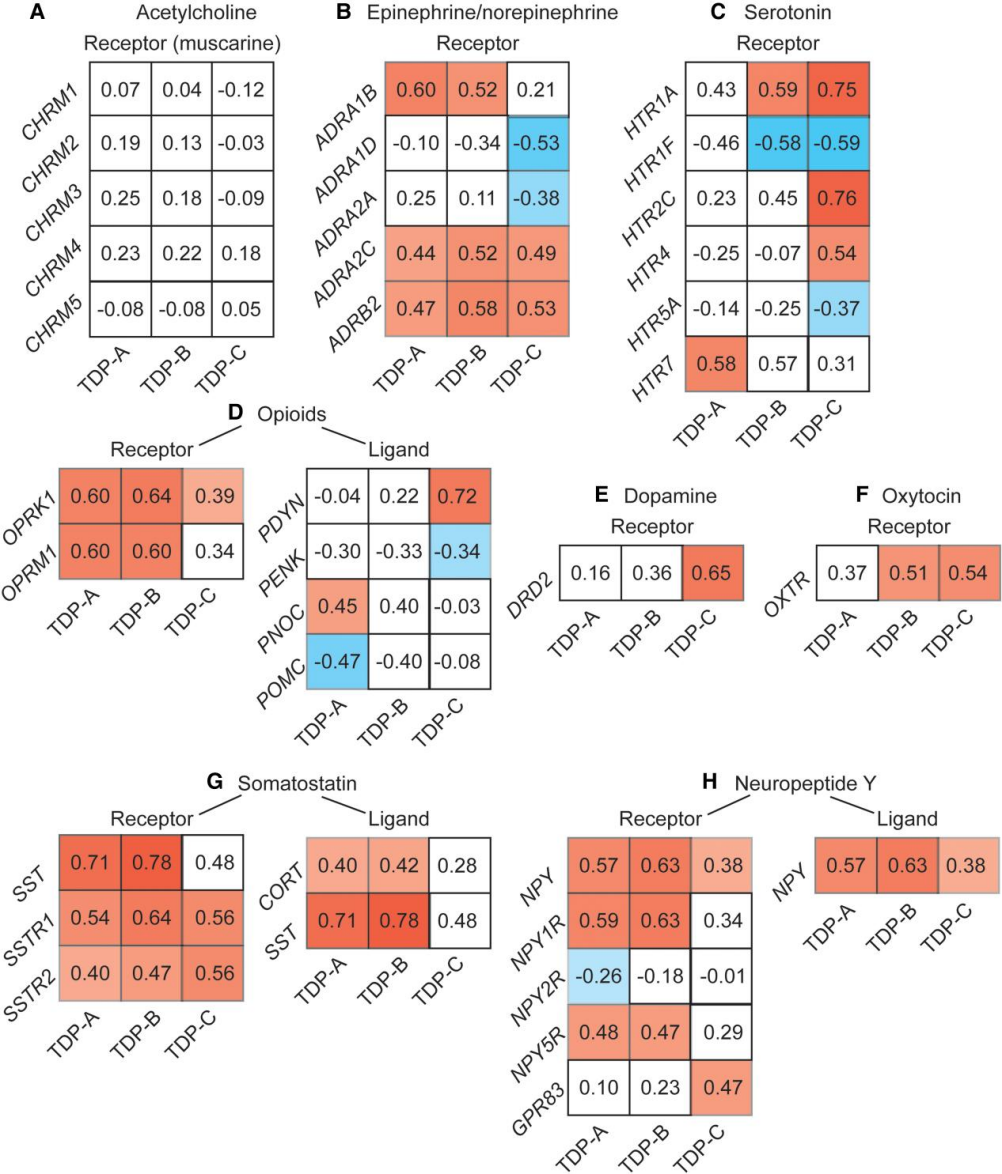
**Genes correlating with atrophy in FTLD are enriched for neuromodulatory terms**. Gene set enrichment analyses using the spatial correlation values between brain gene expression levels and frontotemporal lobar degeneration (FTLD) atrophy severity revealed a Kyoto Encyclopedia of Genes and Genomes (KEGG) neuroligand receptor interaction pathway in common among most FTLD subtypes. Whereas genes associated with acetylcholine neurotransmission (**A**), a system often preserved in FTLD, did, for the most part, not correlate with FTLD atrophy. Shared and unique associations were observed between FTLD subtypes and genes associated with epinephrine (**B**), serotonin (**C**), dopamine (**D**), oxytocin (**E**), opioids (**F**), somatostatin (**G**) and neuropeptide Y receptor and ligand systems (**H**). Matrices reflect the strength and sign of spatial correlation between genes associated with ligand and receptor systems and atrophy maps in FTLD subtypes. White squares reflect genes whose correlation with FTLD atrophy maps did not reach significance based on the bootstrap test correcting for spatial autocorrelation (FDR adjusted *P* < 0.05).

To better understand the biological function of FTLD atrophy-correlated genes, we performed gene set enrichment analyses^50^ against Gene Ontology and KEGG.^51^ While no gene ontology was considered enriched, the gene set enrichment analysis revealed only one KEGG pathway, associated with neuroactive ligand-receptor interactions (has04080),^52^ shared across FTLD-TDP and FTLD-Pick subtypes. This KEGG pathway consisted of common and unique associations between FTLD atrophy-correlated genes and major classes of neuromodulatory and homeostatic neurotransmitter systems (Fig. 2 and Supplementary Fig. 5). Genes associated with acetylcholine receptors did not, for the most part, correlate with atrophy in any FTLD subtype (Fig. 2A), as expected based on the lack of a cholinergic deficit in FTLD.^60^ Conversely, genes associated with receptors within the epinephrine/norepinephrine, opioid, somatostatin and neuropeptide Y systems correlated across most FTLD subtypes (Fig. 2B and F–H). Some receptor systems showed more specific associations with FTLD subtypes, as in the case of serotonin and dopamine to TDP-C (Fig. 2C and D), or oxytocin to TDP-B, TDP-C and Pick's disease (Fig. 2E). The lack of associations for CBD is likely attributable to a low number of genes associated with atrophy in this subtype and the high spatial autocorrelation of its atrophy map.

### Genes correlated with FTLD atrophy are enriched for HAR genes

**Figure 3:**
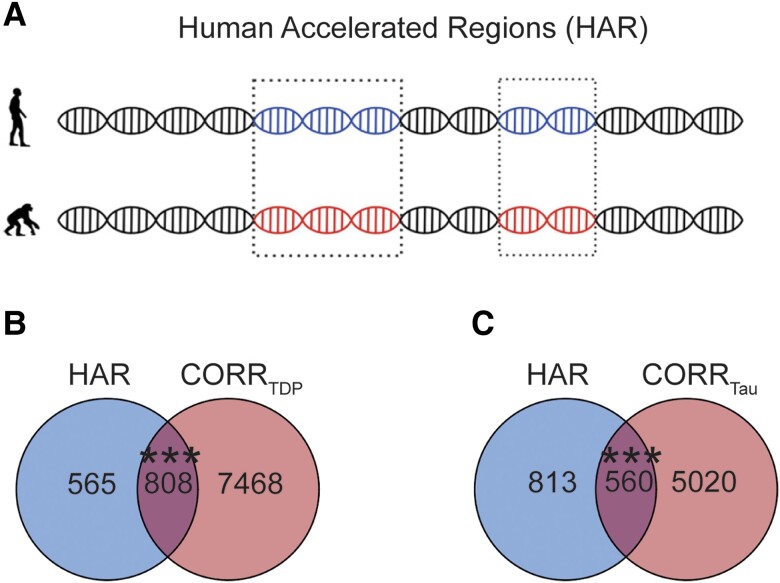
**Overlap between FTLD atrophy correlated genes and HAR genes**. (**A**) HARs are conserved genomic loci which have undergone accelerated divergence in the human evolutionary lineage. (**B**) Genes correlating with atrophy in FTLD-TDP had 808 genes in common with HAR genes, (**C**) while genes correlating with atrophy in FTLD-tau had 560 genes in common with HAR genes. Both overlaps were unlikely to occur by chance when compared to a background set of brain-expressed genes. ***P < 0.0005. (**A**) adapted with permission from Doan *et al*.^7^ and Wei *et al*.^45^ FTLD = frontotemporal lobar degeneration.

First, we identified 1373 brain-expressed HAR genes derived from a comparative genomic study of conserved loci with elevated divergence in humans versus chimpanzees and other mammals (Fig. 3A and Supplementary material).^7^ We next assessed the overlap between brain-expressed HAR genes and genes whose expression patterns correlated with atrophy in FTLD using Fisher's exact test. Atrophy-correlated genes were enriched for HAR genes in both FTLD-TDP (808 genes, *P* < 0.0005, Fig. 3B) and FTLD-tau (560 genes, *P* < 0.0005, Fig. 3C). Of the 808 FTLD-TDP atrophy-correlated HAR genes, 339 correlated negatively and 470 correlated positively with atrophy (*ADCYAP1* correlated both positively and negatively with atrophy in at least one FTLD-TDP subtype). Of the 560 FTLD-tau atrophy-correlated HAR genes, 202 correlated negatively and 358 correlated positively with atrophy. Of the HAR genes correlating with at least one FTLD subtype, 283 were unique to FTLD-TDP, 525 were shared between FTLD-TDP and FTLD-tau (Supplementary Fig. 6A), with only 35 HAR genes correlating uniquely with at least one FTLD-tau subtype. The association between HAR genes and atrophy-correlated genes in FTLD-TDP and FTLD-tau remained significant also after applying more stringent statistical thresholds to derive atrophy-correlated genes (FDR adjusted *P* < 0.01; Supplementary Fig. 7).

To evaluate the specificity of the overlap between HAR genes and FTLD atrophy-correlated genes, we leveraged a publicly available W-score map of cortical atrophy from 147 amyloid- and tau-confirmed patients with Alzheimer's disease-type dementia (henceforth, AD).^48^ This analysis revealed an overlap between HAR genes and AD atrophy-correlated genes, but this overlap did not reach significance and was statistically smaller that the overlap between HAR genes and random FTLD-TDP and FTLD-tau atrophy-correlated gene lists of same size as the AD atrophy-correlated genes (Supplementary Fig. 8).

### FTLD-TDP atrophy-correlated genes are linked to TDP-43 regulated genes

**Figure 4:**
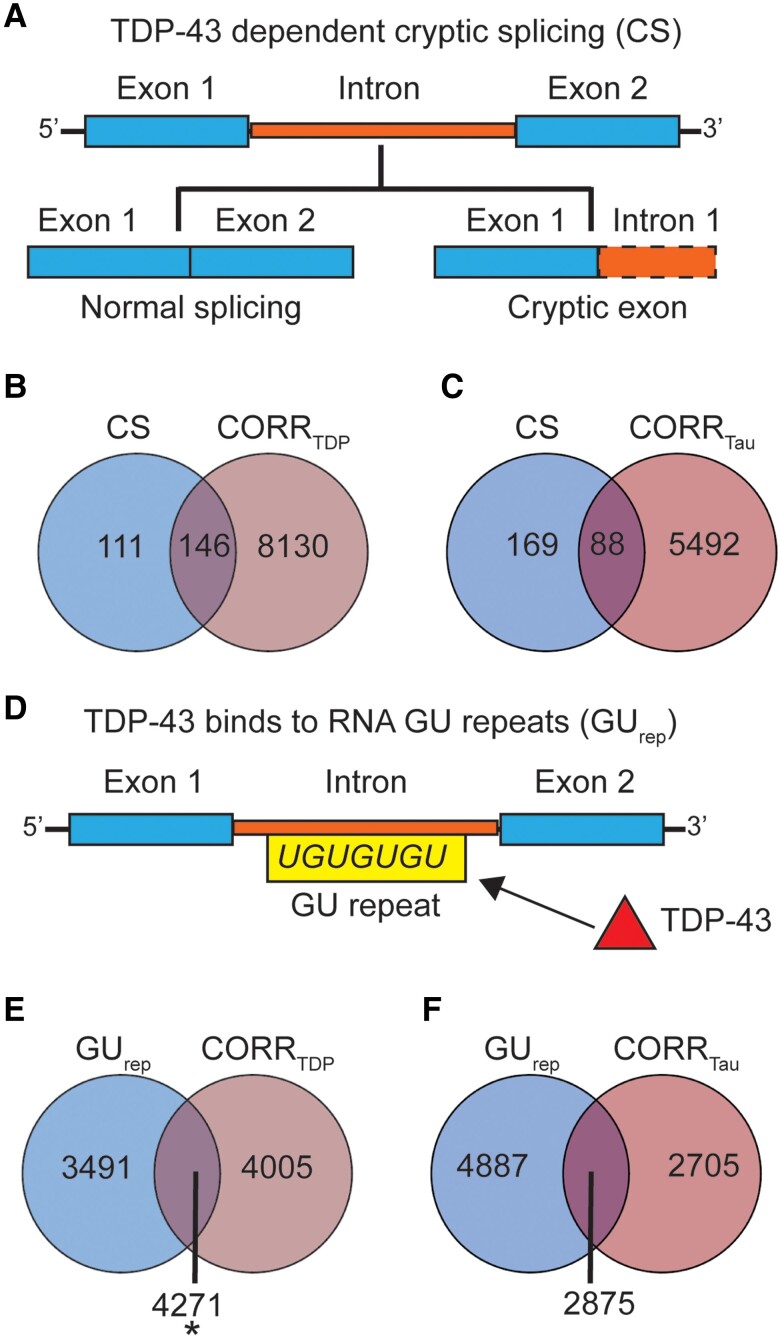
**Overlap between FTLD atrophy correlated genes and genes regulated by TDP-43**. (**A**) Cryptic splicing (CS) genes are genes that incorporate novel intronic RNA into mature mRNA when TDP-43 is knocked down experimentally or depleted from diseased neuronal nuclei. (**B**) One hundred and forty-six genes were shared between cryptic splicing genes and genes correlating with atrophy in FTLD-TDP. This overlap did not reach significance (*P* = 0.11). (**C**) Eighty-eight genes were shared between cryptic splicing genes and genes correlating with atrophy in FTLD-tau, with this overlap being far from significant (*P* = 0.75). (**D**) TDP-43 binds to GU repeats located on the intronic portions of the pre-messenger RNA. GU repeats are ubiquitous among brain-expressed genes, we hence identified a set of 7792 genes with above-median GU repeat content, increasing the likelihood that these genes are regulated by TDP-43. (**E**) Genes (*n* = 4271) were shared between genes with above-median GU repeat content and genes correlating with atrophy in FTLD-TDP. This overlap was significant (*P* = 0.03). (**F**) Genes (*n* = 2875) were shared between genes with above-median GU repeat content and genes correlating with atrophy in FTLD-tau, with this overlap being not significant (*P* = 0.13). *P < 0.05. FTLD = frontotemporal lobar degeneration.

Recent studies have identified a corpus of genes that undergo cryptic splicing upon TDP-43 loss-of-function^24-26^ (Fig. 4A). Given the strong enrichment of FTLD-TDP atrophy-correlated genes for HAR genes, and that humans show non-conserved patterns of TDP-43 related cryptic splicing, we hypothesized that TDP-43 cryptic splicing genes might be enriched among genes correlated with FTLD-TDP (but not FTLD-tau) atrophy. To explore this possibility, we consolidated published and unpublished TDP-43 cryptic splicing gene lists, filtering for human brain-expressed cryptic splicing genes identified using human cell models (216 genes)^25,26^ or patient brain tissues (63 genes^24^; Supplementary Table 3), resulting in 257 unique cryptic splicing genes (Supplementary material). One hundred and forty-six genes were shared between FTLD-TDP atrophy-correlated and cryptic splicing genes, with 82 cryptic splicing genes correlating negatively and 64 correlating positively with atrophy, yet this overlap was not significant (*P* = 0.11, Fig. 4B). To evaluate the biological relevance of the observed trend, we compared the overlap between cryptic splicing genes and FTLD-TDP atrophy-correlated genes to the overlap between cryptic splicing genes and FTLD-tau atrophy-correlated genes. We identified 88 FTLD-tau correlated cryptic splicing genes, of which 51 correlated negatively and 37 correlated positively with atrophy. In contrast to FTLD-TDP, however, this overlap did not approach significance (*P* = 0.71, Fig. 4C). The proportion of cryptic splicing genes overlapping with FTLD-TDP atrophy-correlated genes was significantly higher than the proportion of cryptic splicing genes overlapping with FTLD-tau atrophy-correlated genes [*χ*^2^ (2,514) = 26.39, *P* < 0.0001]. To control these analyses for the smaller number of FTLD-tau atrophy-correlated genes, we randomly re-sampled (5000 times) lists of 5580 FTLD-TDP atrophy-correlated genes (matching the number of FTLD-tau atrophy-correlated genes). This approach revealed a significantly higher overlap with cryptic splicing genes for FTLD-TDP when compared to FTLD-tau atrophy-correlated genes (Supplementary Fig. 9, *P <* 0.05), suggesting that the differential overlap between FTLD-TDP and FTLD-tau atrophy-correlated genes with cryptic splicing genes is not merely a reflection of gene list length. Cryptic splicing genes correlated with at least one FTLD subtype were either unique to FTLD-TDP (63 genes) or shared between FTLD-TDP and FTLD-tau (83 genes) (Supplementary Fig. 6B). Overall, these finding suggest that although both FTLD-TDP and FTLD-tau target brain regions expressing recently evolved genes, FTLD-TDP exhibits a closer relationship to regions that express cryptic splicing genes.

To strengthen the association between TDP-43 transcriptional regulation functions and genes linked to atrophy in FTLD-TDP, we next determined brain-expressed genes with a higher likelihood of being regulated by TDP-43, operationally defined here as having an above-median number of GU repeats, the putative binding site of TDP-43 (Fig. 4D).^24^ This approach yielded 7792 genes containing higher amounts of GU repeats compared to other brain-expressed genes [GU tetramers, median (range) = 324 (46–11 313); GU pentamers, median (range) = 107 (23–4087); GU hexamers, median (range) = 37 (5–1663)]. We found a significant overlap between genes with above-median GU repeat content and genes correlating with atrophy in FTLD-TDP (*P* = 0.03), but not for genes correlating with atrophy in FTLD-tau (*P* = 0.13) (Fig. 4E and F).

### HAR genes overlap with TDP-43 cryptic splicing genes

**Figure 5:**
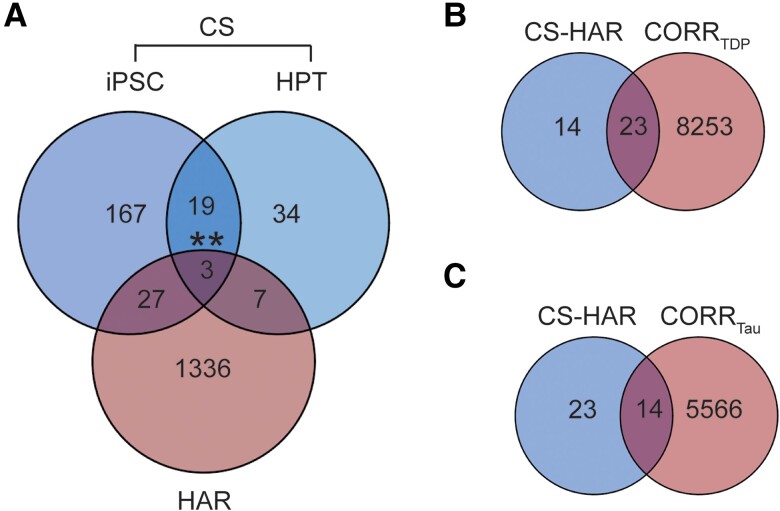
**HAR and cryptic splicing genes overlap**. (**A**) Comparing the human accelerated region (HAR) and cryptic splicing (CS) gene lists, including those derived from induced pluripotent stem cell (iPSC)-derived neurons and from human patient tissues (HPT), 37 total genes were shared. Fisher's exact test was used to determine whether gene sets overlapped at a rate greater than expected by chance. We then explored the overlap between CS-HAR genes and genes correlating with atrophy in FTLD-TDP or FTLD-tau. (**B**) Twenty-three genes were shared between CS-HAR genes and genes correlating with atrophy in FTLD-TDP. This overlap did not reach significance (*P* = 0.17). (**C**) Fourteen genes were shared between CS-HAR genes and genes correlating with atrophy in FTLD-tau, with this overlap not being significant (*P* = 0.98). **P < 0.005. FTLD = frontotemporal lobar degeneration.

Among the TDP-43 CS genes, 37 were also HAR genes (Fig. 5A and Supplementary material), an overlap that was higher than expected by chance (Fisher's exact test). Identified CS-HAR genes included genes linked to social behaviour and language (e.g. *ERC2*, *KIF26B*, *CBLN2*, *SEMA6D*),^61-65^ as well as neurodevelopment and neuropsychiatric diseases (e.g. *CBLN2SPRY3*, *CAMTA1*, *LRP8*).^66-70^ Some CS-HAR genes identified (e.g. *CBLN2*, *ICA1*, *PTPRT* and *PTPRD*) have been shown to accurately distinguish FTLD-TDP post-mortem cortex from non-neurological disease control tissue based on the frontal/temporal cortex expression of cryptic exons.^26^ CS-HAR genes tended to be longer than other brain-expressed genes [*t*(39) = 4.74; *P* < 0.0001], but their length fell within the range found for 85% of brain expressed genes (Supplementary Fig. 10A). CS-HAR genes contained mostly intronic HAR sequences (76%, Supplementary Fig. 10B), followed by intergenic (21%) and exonic (3%) sequences, and cryptic splicing events affecting CS-HAR genes mainly included cryptic exon incorporation^20^ (71%, Supplementary Fig. 10C), consistent with other (non-HAR) cryptic splicing genes. We performed control analyses assessing the overlap between cryptic splicing genes and a set of non-HAR genes matched for length to HAR genes (Supplementary Fig. 11A). This approach yielded an overlap of 42 genes (Supplementary Fig. 11B) [*χ*^2^(2,2726) = 0.41, *P* = 0.52], suggesting that gene size is a confounding factor driving the overlap between cryptic splicing and HAR genes. Therefore, although HAR and TDP-43 cryptic splicing genes overlap, our analyses do not provide evidence for a special, gene length-independent relationship between HAR and TDP-43 cryptic splicing genes.

**Figure 6:**
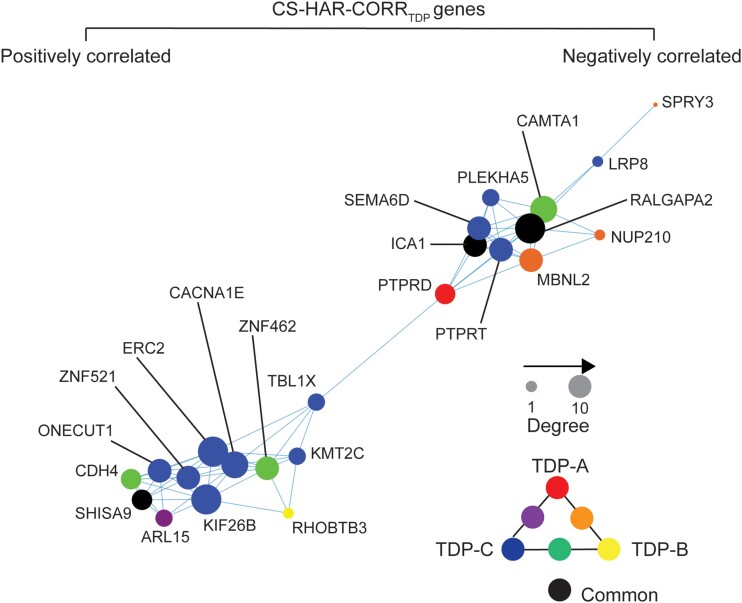
**Co-expression network of CS-HAR genes associated with FTLD-TDP atrophy**. The regional expression covariance of CS-HAR genes associated with FTLD-TDP atrophy was used to generate a topographical network of gene expression. The size of each circle reflects the centrality of each gene in the network, while the colour of the circles reflects the FTLD-TDP subtype whose atrophy pattern correlated with the gene's expression pattern. CS = cryptic splicing; FTLD = frontotemporal lobar degeneration; HAR = human accelerated region.

We next sought to better understand relationships between FTLD atrophy-correlated genes and the 37 CS-HAR genes (Fig. 5A). Of these 37 genes, 23 overlapped with FTLD-TDP atrophy-correlated genes (*P* = 0.17, Fig. 5B) and 14 with FTLD-tau atrophy-correlated genes (*P* = 0.98, Fig. 5C), but these overlaps were not greater than expected by chance. Of the CS-HAR genes correlating with at least one FTLD subtype, 10 were unique to FTLD-TDP and 13 were shared between FTLD-TDP and FTLD-tau (Supplementary Fig. 6C). Accordingly, to generate hypotheses, we examined the relationships among the 23 FTLD-TDP atrophy-associated CS-HAR genes in terms of their co-expression relationships in the healthy brain and their links to specific FTLD-TDP subtypes (Fig. 6). While some genes correlated negatively with atrophy (e.g. *PTPRD*), suggesting lower average expression among FTLD-TDP atrophied regions, other genes correlated positively (e.g. *ERC2*), reflecting higher expression among targeted regions. Some FTLD-TDP subtype-specific associations emerged, such as *PTPRD* with TDP-A, *RHOBTB3* with TDP-B, or *ERC2* with TDP-C, while other genes, such as *RALGAPA2*, *ICA1* and *SHISA9*, were shared among all FTLD-TDP subtypes.

### Expression of cryptic splicing and HAR genes varies across FTLD-TDP subtypes and patient epicentres

**Figure 7:**
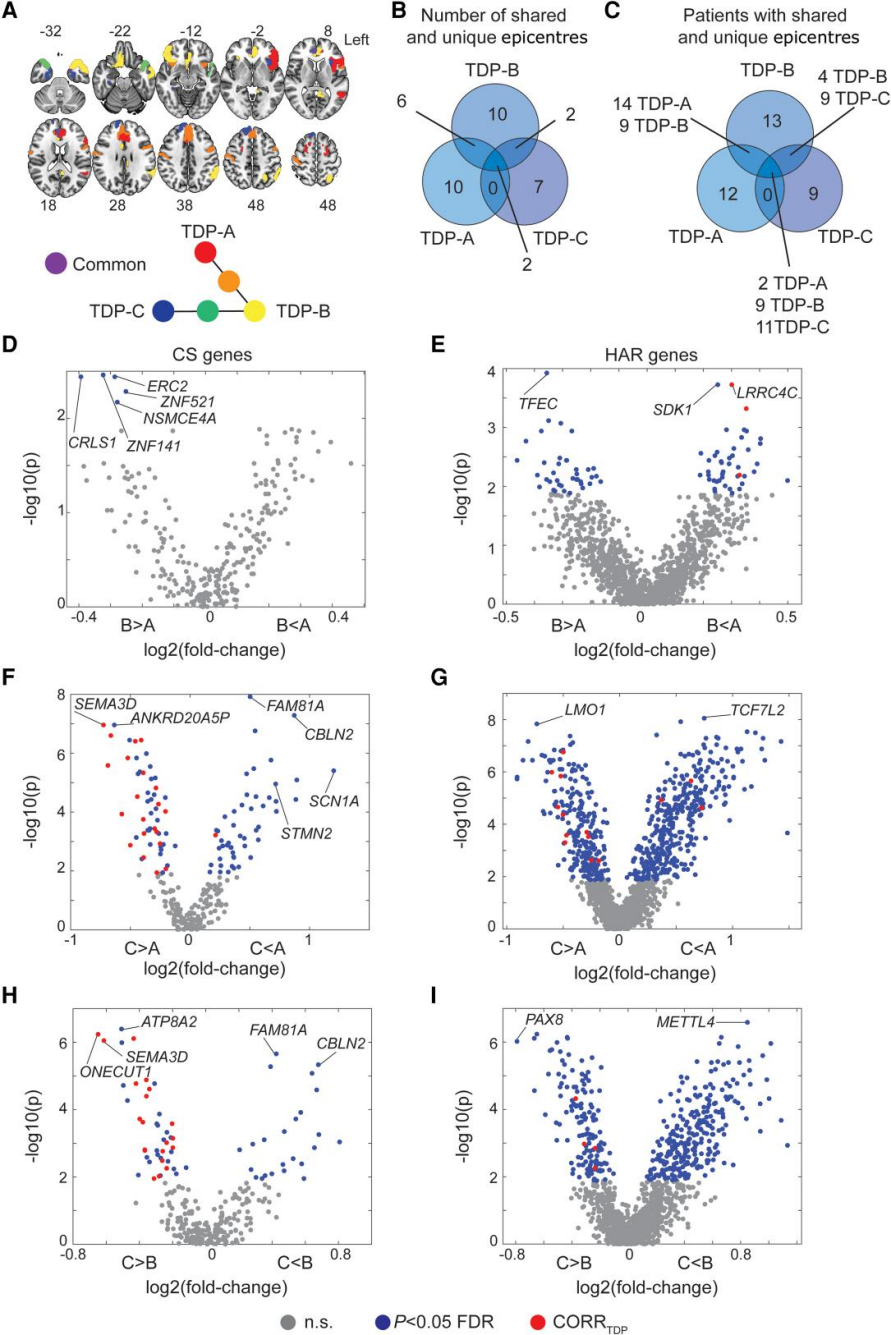
**Average expression of cryptic splicing and HAR genes in FTLD-TDP disease epicentres**. (**A**) Neuroanatomical distribution of FTLD-TDP subtype unique and shared epicentres. (**B**) Number of epicentres unique or shared across FTLD-TDP subtypes. (**C**) Number of patients with FTLD-TDP having unique or shared epicentres. Epicentres' average expression of cryptic splicing genes compared between (**D**) TDP-A and TDP-B, (**E**) TDP-A and TDP-C, and (**F**) TDP-B and TDP-C. Epicentres' average expression of human accelerated region (HAR) genes compared between (**G**) TDP-A and TDP-B, (**H**) TDP-A and TDP-C, (**I**) TDP-B and TDP-C. Grey circles depict genes with average expression within epicentres that do not significantly (n.s.) differ across FTLD-TDP subtypes, blue circles depict genes with average expression within epicentres that significantly differ (FDR adjusted *P* < 0.05) across FTLD-TDP subtypes, red circles depict genes with average expression within epicentres that significantly differ (FDR adjusted *P* < 0.05) across FTLD-TDP subtypes and also positively correlate with atrophy in the FTLD-TDP subtype where they are more expressed. FTLD = frontotemporal lobar degeneration.

The preceding analyses used group-level methods to identify brain-expressed HAR and cryptic splicing genes associated with cortical grey matter atrophy in FTLD subtypes, but there is also prominent anatomical heterogeneity within each subtype,^71^ leading us to explore a more individualized, patient-tailored approach. Neurodegenerative diseases begin within specific vulnerable and network-anchoring regions, which we have termed ‘epicentres’,^54,56^ before spreading to other areas along large-scale brain network connections.^4,56,72^ In previous work, we introduced a method for identifying patient-specific epicentres, which enhanced network-based predictions of longitudinal atrophy progression in FTLD.^54,56^ This approach uses task-free functional MRI acquired in a normative sample to estimate each brain region's connectivity to all other brain regions (Supplementary Fig. 12A) and correlates these connectivity maps with an individual patient's atrophy pattern to identify the region (i.e. epicentre) whose normative connectivity most resembles the atrophy pattern.^54,56,57^ Based on this approach, we identified a single best-fit epicentre for each patient, focusing on FTLD-TDP given its specific relationship to cryptic splicing (Fig. 7A). Frequency maps revealed common epicentres in TDP-A, including the pregenual and subgenual anterior cingulate cortices and the anterior insula, with the anterior cingulate being the most common epicentre. The left anterior insula was the most common epicentre in TDP-B, which showed additional epicentres in the anterior cingulate and temporal pole. In TDP-C, the left entorhinal cortex was the most common epicentre, with additional epicentres in adjacent temporal polar regions (Supplementary Fig. 12B–D). While many epicentres were shared across subtypes (e.g. the left anterior cingulate cortex), several were associated with just one FTLD-TDP subtype, such as the left inferior frontal gyrus in TDP-A, the left angular gyrus in TDP-B, or the left fusiform gyrus with TDP-C (Fig. 7A). Overall, despite each FTLD-TDP subtype providing 7–10 uniquely associated epicentres (Fig. 7B), a substantial proportion of patients shared epicentres across subtypes (Fig. 7C), suggesting that any influence of gene expression on subtype-associated regional vulnerability is not likely to be deterministic.

To formulate hypotheses regarding potential HAR- and CS-related genes and pathways whose expression might influence the onset of FTLD-TDP in different regions across different FTLD-TDP subtypes, we then compared the average expression of HAR genes or TDP-43 cryptic splicing genes across FTLD-TDP subtype epicentres by using Wilcoxon signed-rank tests (FDR adjusted *P <* 0.05). Resulting *P-*values were plotted against the cross-fold change, reflecting higher or lower gene expression in epicentres of one FTLD-TDP subtype versus another (Fig. 7D–I). These analyses revealed genes more highly expressed in regional epicentres found in TDP-B compared to TDP-A, such as cryptic splicing gene *ERC2* and HAR gene *TFEC* (Fig. 7D and E), and genes more highly expressed in epicentres for TDP-A versus TDP-B, such as HAR genes *LRRC4C* and *SDK1* (Fig. 7E). When comparing TDP-A to TDP-C, the cryptic splicing gene *STMN2* was significantly more expressed in regional epicentres found in TDP-A, while the cryptic splicing gene *SEMA3D* was more expressed in regional epicentres found in TDP-C (Fig. 7F). The HAR gene *LMO1* was significantly more expressed in epicentres of TDP-C, while the HAR gene *TCF7L2* was more expressed in epicentres of TDP-A (Fig. 7G). When comparing TDP-B to TDP-C, the cryptic splicing gene *CBLN2* and the HAR gene *METTL4* showed significantly higher expression among disease epicentres derived from TDP-B, while the cryptic splicing gene *ONECUT1* and the HAR gene *PAX8* were more highly expressed in epicentres of TDP-C (Fig. 7H and I).

## Discussion

The integrative anatomical biology data presented here identified overlapping and distinct genes associated with cortical atrophy in FTLD-TDP and FTLD-tau. Both gene sets were enriched for HAR genes, providing novel comparative transcriptomic evidence that FTLD selective vulnerability, regardless of the underlying molecular pathology, relates to genes that have undergone recent evolution as humans diverged from chimpanzees. Although HAR genes have been associated with neuropsychiatric and neurodevelopmental disorders,^7^ FTLD is the first neurodegenerative disease that has been directly linked to regional patterns of HAR gene expression. Human studies leveraging chromatin interaction profiles in the fetal and adult cortex have revealed that specific cell types and laminae involved in cerebral cortical expansion are enriched for HAR elements.^73^ These findings echo neuroimaging studies showing that the expression of HAR genes in the healthy human cerebral cortex is highest among brain networks that have undergone rapid evolutionary expansion in humans.^45^ These recently expanded higher-order networks overlap with systems supporting language and social-emotional functions, the same systems that undergo early and progressive neurodegeneration in FTLD.^71^ Here, the expression of atrophy-associated HAR and non-HAR genes was either positively or negatively associated with FTLD atrophy. Genes showing a positively associated gradient are likely to be highly expressed, in the healthy brain, in regions subject to FTLD atrophy. Dependence on these genes could contribute to FTLD regional vulnerability, especially if protein expression is diminished, for example by TDP-43 loss-of-function and associated mis-splicing. Conversely, genes displaying a negative gradient are probably expressed at lower levels in the healthy brain among FTLD-atrophied regions, with neurodegeneration further compromising already low levels of expression. FTLD atrophy-associated genes were characterized by common and unique associations with neuromodulatory and homeostatic transmitter systems affected in FTLD.^74^ Dopaminergic, norepinephrinergic and serotonergic imbalances are common in FTD, while the acetylcholine system remains relatively intact.^60^ Common antidepressants, such as selective serotonin reuptake inhibitors, have been shown to improve the behavioural symptoms of FTD,^60^ reinforcing the notion of neuromodulatory imbalances in FTLD. Our analyses further revealed that FTLD-TDP atrophy-correlated genes are slightly enriched for genes that are targets of TDP-43 cryptic splicing repression, significantly more so than FTLD-tau atrophy-correlated genes. This observation, though tentative, suggests a potential FTLD-TDP-specific mechanism whereby incipient TDP-43 loss-of-function focuses vulnerability on specific brain networks by depleting regional levels of TDP-43 CS genes.

Several lines of evidence derived from animal^75,76^ and cellular models,^77^ as well as human neuroimaging^54,56^ and tissue studies,^30^ support the notion that neuropathological changes start within vulnerable brain regions, often referred to as epicentres, before spreading via network pathways.^78,79^ In this context, our study not only shows that the expression of HAR and cryptic splicing genes resembles atrophy patterns in FTLD-TDP but also that the expression of these genes within putative epicentres differs among FTLD-TDP subtypes. Our findings raise the possibility that the regional expression of specific HAR and TDP-43 cryptic splicing genes may predispose local circuits to cultivate specific FTLD-TDP subtypes, each characterized by a distinct TDP-43 aggregation pattern.^80^ After accumulating within vulnerable epicentres, TDP-43 aggregates could then spread to other brain regions via functional and structural connectivity pathways. Histopathological studies suggest that neurodegenerative diseases may start within susceptible cell-types harboured within vulnerable brain regions.^4,14,81,82^ For example, von Economo neurons and fork cells have been shown to be selectively targeted by TDP-43 depletion and inclusions in bvFTD due to FTLD-TDP.^83^ These large, elongated neuron types are almost exclusively found within the anterior cingulate and frontoinsula of large-brained, highly social mammals^84^—including humans, greater apes, elephants and cetaceans—raising the possibility that these neurons contribute to circuits linked to the emergence of complex social behaviours.^84^ Further, a recent neuroimaging genomics study revealed that atrophied regions in FTD genetic mutation carriers express astrocyte and endothelial cell-related genes.^47^ Future histopathological studies and cell-type enrichment analyses could shed light on how expression levels of HAR and cryptic splicing genes within vulnerable neuronal, such as von Economo neurons, interact with glial cells and contribute to the emergence of distinct FTLD-TDP subtypes.

Our findings should be interpreted in light of several methodological considerations. First, our study used average brain transcriptomic data derived from the AHBA, which is based on bulk tissue microarray data from the brains of six donors. Both hemispheres were collected in only two of six donors (with the remainder providing only the left). Future studies leveraging transcriptomic data derived from larger samples are needed to confirm the transcriptomic correlates of regional grey matter atrophy in FTLD. Moreover, we used a W-model approach to correct for the influence of several confounding factors on grey matter atrophy, including scanner type. It is possible that residual influence from scanner type survived our approach, which could be mitigated in future studies by applying more sophisticated harmonization methods.^85^ Further, our study focused on cortical gene expression, omitting subcortical areas despite the relevance of the striatum, thalamus and other subcortical areas in FTLD.^86^ Gene expression levels from bulk tissue microarray dramatically differ depending on whether these are extracted from cortical or subcortical areas, with most studies, including ours, analysing either one set of structures or the other.^42,45^ The overlap we observed between HAR and cryptic splicing genes, though interesting, appears to be driven primarily by gene length. This makes sense given that longer genes may be implicated in multiple processes including brain development and neurodegenerative diseases.^87,88^ Further studies are needed to assess whether altered sequences within HAR genes mechanistically interact with genomic regions regulated or bound by TDP-43. Finally, our study focused on cryptic splicing genes identified in FTLD-TDP human post-mortem tissue data^24^ or iPSC-derived neuronal cell lines,^25,26^ omitting those identified in HeLa cell lines.^89^ Although this choice was intended to enhance biological confidence regarding the neural relevance of these genes, future studies may expand the TDP-43 ‘cryptic spliceosome’ to explore a more exhaustive gene set.

## Supplementary Material

awae205_Supplementary_Data

## Data Availability

In house Python 2.7.16 (https://www.python.org/), R 4.1.2 (https://www.r-project.org/) and MATLAB R2021a (https://www.mathworks.com/products/matlab.html) scripts were used for the analyses. W-score maps averaged for each FTLD subtype are publicly available on NeuroVault^90^ (https://identifiers.org/neurovault.collection:16591). The gene lists used to generate the findings are available as Supplementary material. Further data that support the findings of this study are available on request from the corresponding author. Correspondence should be directed to Lorenzo Pasquini or William W. Seeley.
