# Supplementary material for: Frontotemporal lobar degeneration targets brain regions linked to expression of recently evolved genes
Source: Brain. 2024 Jun 28;147(9):3032–47. doi: 10.1093/brain/awae205 (PMC11370792; doi:10.1093/brain/awae205)
Supplement: awae205_Supplementary_Data [file awae205_supplementary_data.zip › brain-2023-02221-File010.pdf]

Supplementary Materials for "FTLD targets brain regions linked to expression of recently evolved genes"

Lorenzo Pasquini[1,2*], Felipe L. Pereira[1], Sahba Seddighi[3], Yi Zeng[4], Yongbin Wei[5], Ignacio Illán-Gala[1,6,7], Sarat C. Vatsavayai[1], Adit Friedberg[1,6], Alex J. Lee[1], Jesse A. Brown[1], Salvatore Spina[1], Lea T. Grinberg[1,8], Daniel W. Sirkis[1], Luke W. Bonham[1,9], Jennifer S. Yokoyama[1,9], Adam L. Boxer[1], Joel H. Kramer[1], Howard J. Rosen[1], Jack Humphrey[10], Aaron D. Gitler[4], Bruce L. Miller[1], Katherine S. Pollard[11,12,13,14], Michael E. Ward[3], William W. Seeley[1,8*]

Supplementary Materials

- Supplementary Findings: 1
- Supplementary Figures: 12
- Supplementary Tables: 3
- Supplementary Data: 1
- Supplementary Appendix: 1

Supplementary Findings:

To further evaluate the potential influence of scanner type on our findings, we performed several analyses to assess whether the W-score model effectively controlled for scanner type in the generation of patients' W-score maps. First, individual W-score maps were vectorized and concatenated across subjects, resulting in a patient x regions-of-interest matrix (***Supplementary Figure S1***). A principal component analysis was performed on the patient x regions-of-interest W-score matrix, which revealed a primary component (PC-1) explaining 55% of the variance in the data, and a secondary component (PC-2) explaining 10% of the data. We first ran a linear regression model, with dummy variables for scanner type and FTLD subtype predicting PC-1 scores. This model revealed a significant association between PC-1 and FTLD-Pick (β=11.5, p<0.0005), TDP-A (β=10.6, p<0.006), and TDP-B (β=6.7, p<0.02), but no significant association to TDP-C or scanner type. We next ran a second linear regression model, with dummy variables for scanner type and FTLD subtype predicting PC-2 scores. This model revealed a significant association between PC-2 and FTLD-Pick (β=1.8, p<0.05), FTLD-TDP-B (β=2.8, p<0.001), FTLD-TDP-C (β=11.0, p<0.0001), and 1.5 T scanner type (β=1.6, p<0.05). Having established that PC-2 was associated with FTLD-subtype, and, to a weaker extent, also with 1.5 T scanner type, we next investigated whether FTLD subtypes were unevenly distributed across scanner-types. A χ^2 test revealed a significant interaction between scanner type and FTLD subtype (χ^2 [3, 5] = 24.77, p < 0.02) (***Supplementary Table S2***). Having established a significant interaction between scanner type and FTLD subtype, we next investigated the association of PC-1 with FTLD subtype and scanner type by modeling an interaction term between the predictor variables. In this model, PC-1 scores were not significantly predicted by any variable. We finally investigated the association of PC-2 with FTLD-subtype and scanner type by modeling an interaction term between the predictor variables. In this model, PC-2 scores were significantly predicted by FTLD-TDP-C only (β=10.5, p<0.0001). Taken together, these findings suggest a negligeable influence of scanner-type on estimated W-score maps.

Supplementary tables, figures, and legends:

**Age at MRI scan in years**	69.3 (8.8)
**Years of education**	17.2 (2.3)
**Sex (female/male)**	239/158
**Handedness (ambidextrous:left:right)**	0:43:354
**Scanner type (1.5T:Trio:Prisma:4T)**	58/144/140/52
**Total intracranial volume in liters**	1.4 (0.1)

Supplementary Table S1. Demographic characteristics of the 397 healthy older adults used to generate W-score maps.

	**TDP-A**	**TDP-B**	**TDP-C**	**CBD**	**Pick's disease**
**1.5T**	13	9	21	16	14
**4T**	3	7	3	4	3
**Trio**	12	16	5	24	10
**Prisma**	0	3	0	1	0

Supplementary Table S2. Distribution of FTLD-subtypes across the four scanner types. A χ^2 test revealed a significant interaction between scanner-type and FTLD-subtype (χ^2 [3, 5] = 24.77, $p <$ 0.02).

**Gene list**	**Number of genes**	**Source**
Brain-expressed genes	15,656	Allen Human Brain Atlas
HAR genes	2,164	Doan et al., *Neuron* 2019
CS genes 1: FTLD-TDP *postmortem* tissue	66	Ma et al., *Nature* 2022
CS genes 2: iPSC derived neurons study I	107	Brown et al., *Nature* 2022
CS genes 3: iPSC derived neurons study II	233	Sahba et al., *bioRxiv* 2023
FTLD-TDP atrophy-correlated genes (merged across TDP-A, B, and C)	8,276	Structural MRI of 92 patients with autopsy confirmed FTLD-TDP
FTLD-tau atrophy-correlated genes (merged across Pick's and CBD)	5,580	Structural MRI of 72 patients with autopsy confirmed FTLD-tau

Supplementary Table S3. Raw numbers of gene lists involved in the analyses and their sources.

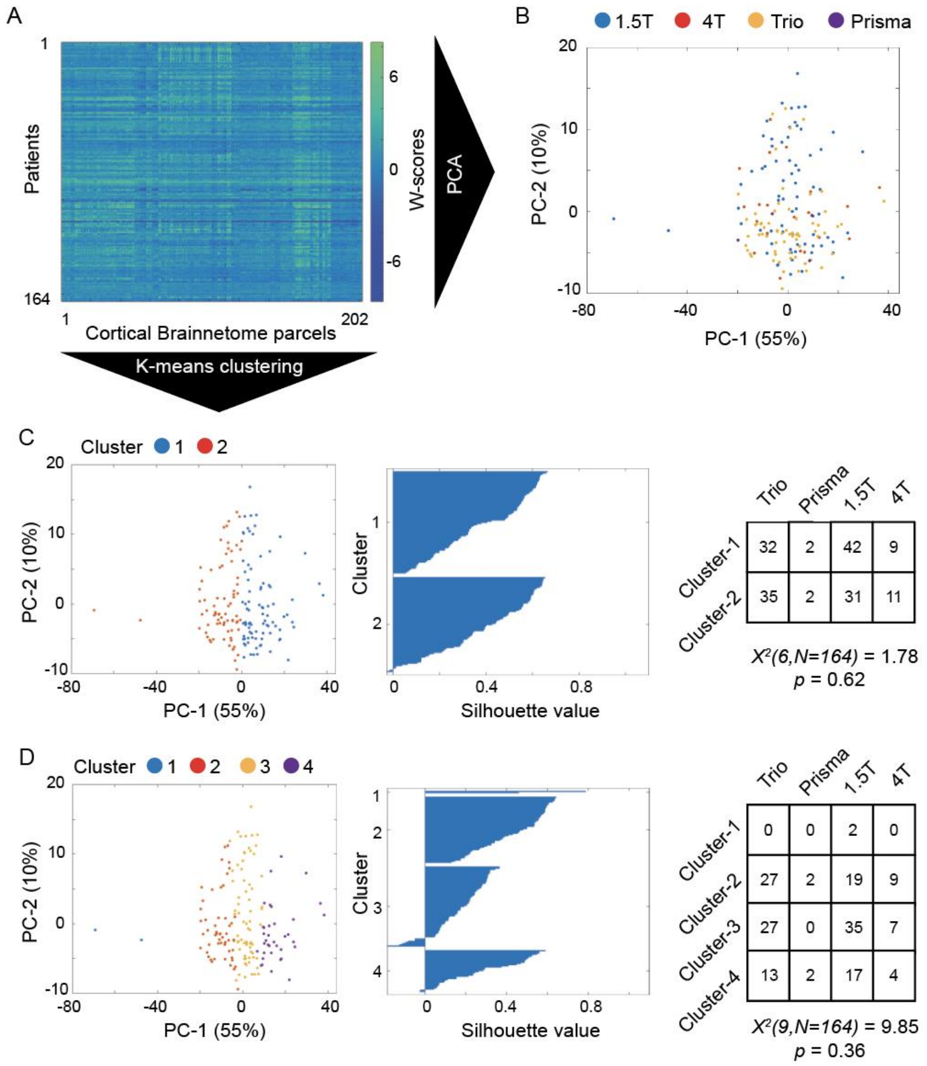

Supplementary Figure S1. The W-score model effectively mitigates the impact of scanner type. (A) Individual patients W-score maps were vectorized and concatenated across subjects, resulting in a patient x regions-of-interest matrix. **(B)** A principal component analysis was performed on the patient x regions-of-interest W-score matrix, which revealed a primary component (PC-1) explaining 55% of the variance in the data. Unsupervised k-means clustering was performed to cluster patients based on their regional W-score values, once by using a solution of 2 clusters (optimal partition based on silhouette plots) **(C)**, and once using a solution with 4 clusters (as many clusters as different scanner types) **(D)**. K-means was performed using Euclidean distance, 10 repetitions, and 10,000 iterations per repetition. X^2 tests were used to assess whether there was any significant relationship between scanner identity of each patient and assigned

cluster. No significant relationship was observed between scanner type and any of the clustering solutions.

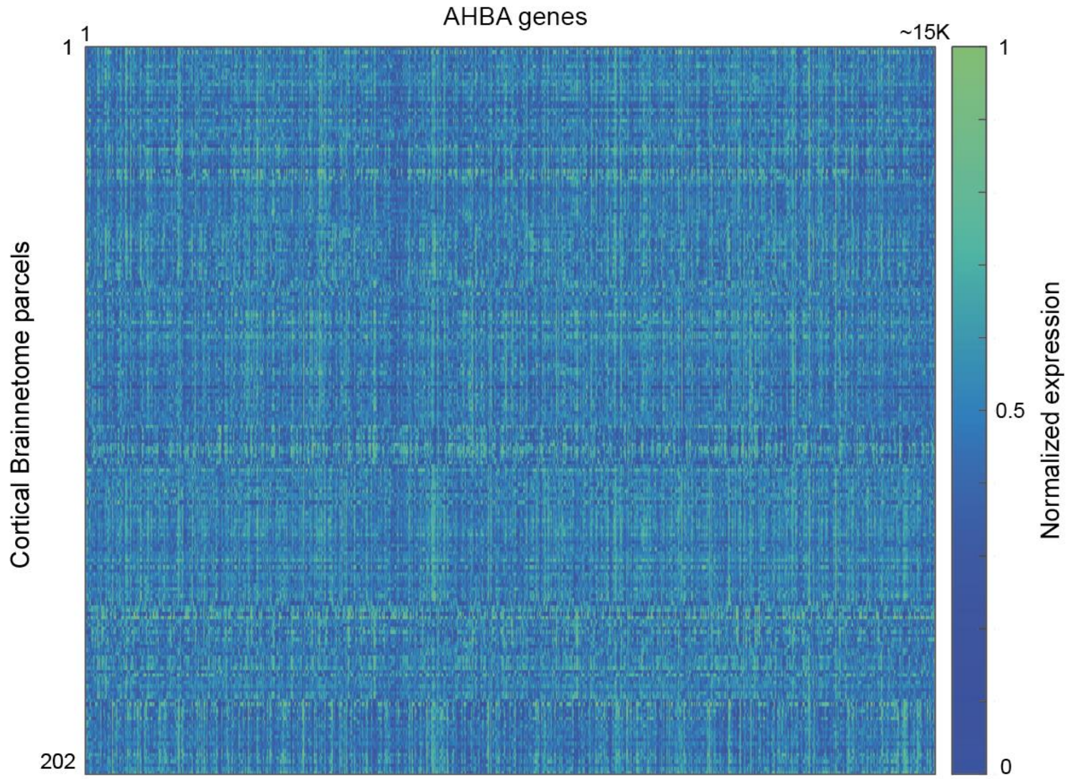

Supplementary Figure S2. Normative gene expression across cortical parcels of the Brainnetome atlas. (A) Heat matrix reflecting the normative expression of AHBA-derived brain genes within cortical parcels of the Brainnetome atlas. Color bar reflects normalized expression values (0-1).

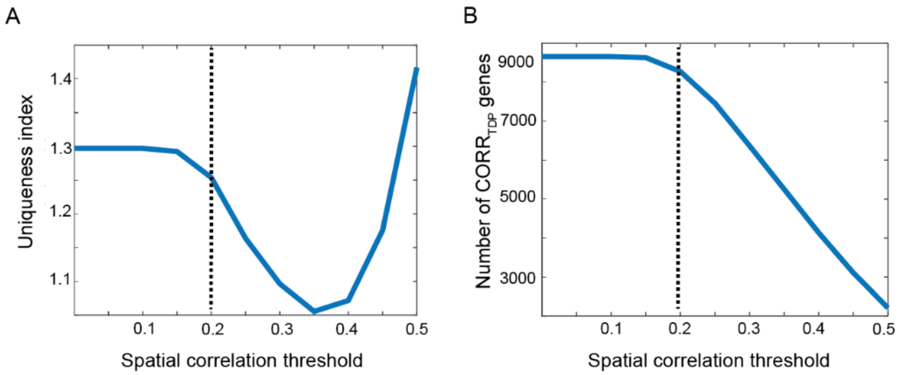

Supplementary Figure S3. Spatial correlation threshold maximizes uniqueness and gene list length. To remove spurious correlations from further analyses, we derived a uniqueness index reflecting the ratio between genes uniquely correlating with single FTLD-TDP subtypes and genes correlating with two or more FTLD-TDP subtypes. The optimal correlation threshold was chosen to **(A)** maximize the uniqueness index and **(B)** the number of genes correlating with FTLD-TDP subtypes.

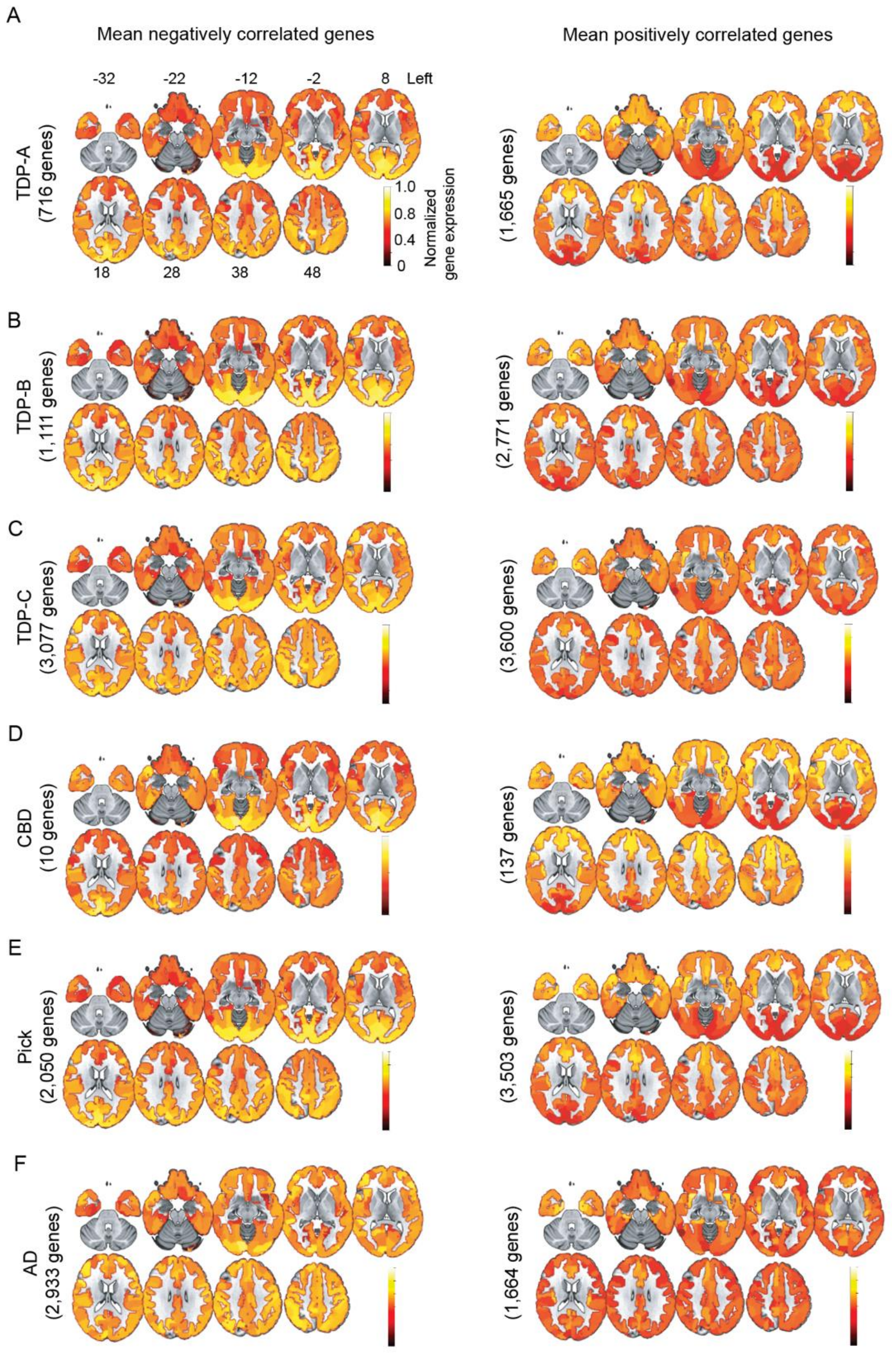

Supplementary Figure S4. Average regional expression of positively and negatively atrophy-correlated genes. Mean regional expression levels for genes negatively (on the left) and positively (on the right) correlated with atrophy in FTLD-TDP-A **(A)**, FTLD-TDP-B (**B**), FTLD-TDP-C (**C**), FTLD-CBD (**D**), FTLD-Pick (**E**), and AD-type dementia (**F**). Warmer colors reflect higher

averaged gene expression, as indicated by the color bar showing normalized levels of gene expression. Number in brackets indicate number of genes correlating either positively or negatively with each neurodegenerative disease subtype. The left hemisphere is shown on the right. Note high mean expression of negatively correlated genes in regions typically spared by FTLD and AD, such as the visual cortices. Conversely, increased expression of positively correlated genes is observed in the insula and anterior cingulate for FTLD-subtypes, and in the medial temporal lobes for AD.

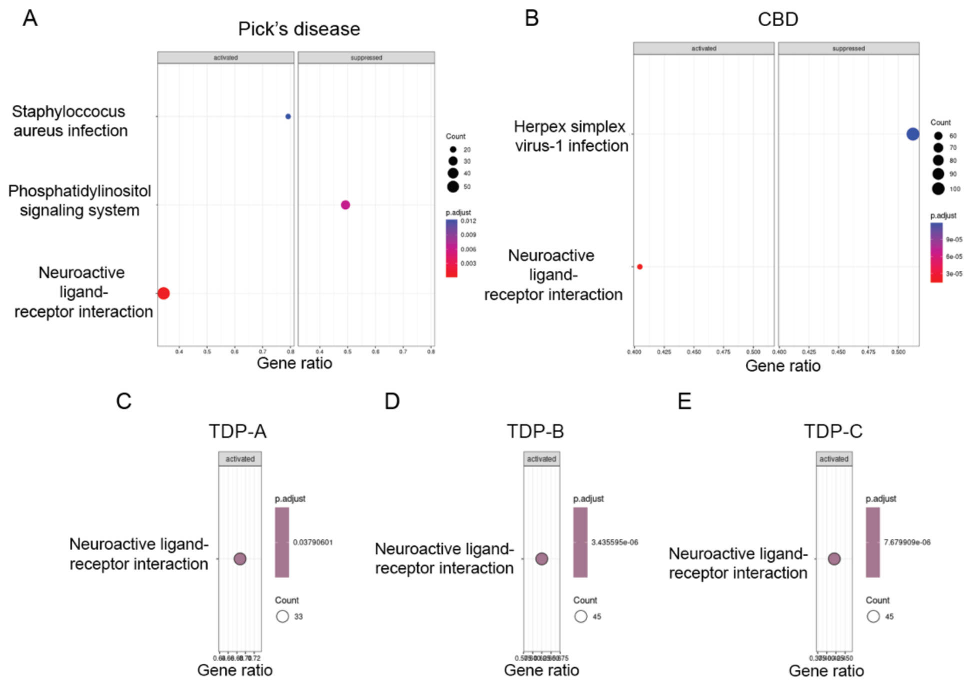

Supplementary Figure S5. KEGG analysis terms and pathways for individual FTLD-subtypes. Terms and pathways from the KEGG analysis for **(A)** Pick's disease, **(B)** CBD, **(C)** TDP-A, **(D)** TDP-B, **(E)** and TDP-C. Neuroactive ligand-receptor interaction was a significant term found across all FTLD-subtypes. Bars reflect adjusted *p*-values; circle size reflects number of genes associated with each term.

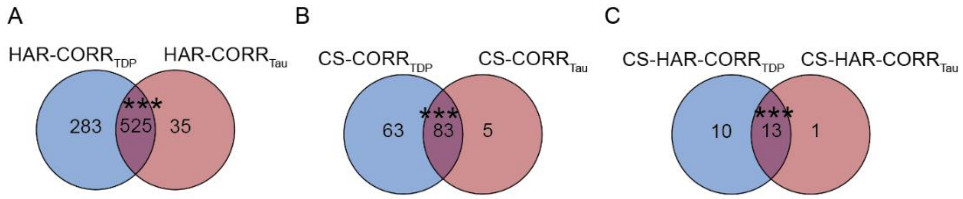

Supplementary Figure S6. Genes correlating with atrophy in FTLD-TDP overlap with genes correlating with atrophy in FTLD-tau. Overlaps between **(A)** HAR genes correlating with atrophy in FTLD-TDP and with atrophy in FTLD-tau; **(B)** CS genes correlating with atrophy in FTLD-TDP and with atrophy in FTLD-tau; and **(C)** CS-HAR genes correlating with atrophy in FTLD-TDP and with atrophy in FTLD-tau. Fisher's exact tests were performed using the totality of brain-expressed HAR genes as background (for A and C) or the totality of brain-expressed CS genes as background (for B). *** $p < 0.005$

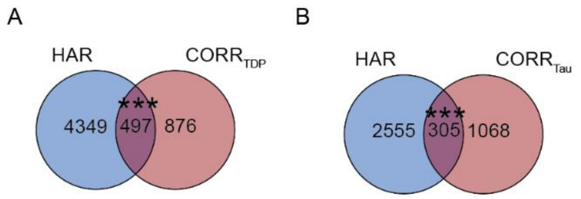

Supplementary Figure S7. Overlap between HAR genes and FTLD atrophy-correlated genes derived using a more stringent statistical threshold. Lists of FTLD atrophy-correlated genes were derived using a more stringent statistical threshold (FDR adjusted $p < 0.01$). **(A)** Using these new gene lists revealed that genes correlating with atrophy in FTLD-TDP had 497 genes in common with HAR genes, **(B)** while genes correlating with atrophy in FTLD-tau had 305 genes in common with HAR genes. Both overlaps were unlikely to occur by chance when compared to the AHBA background set of brain-expressed genes. ***$p < 0.0005$

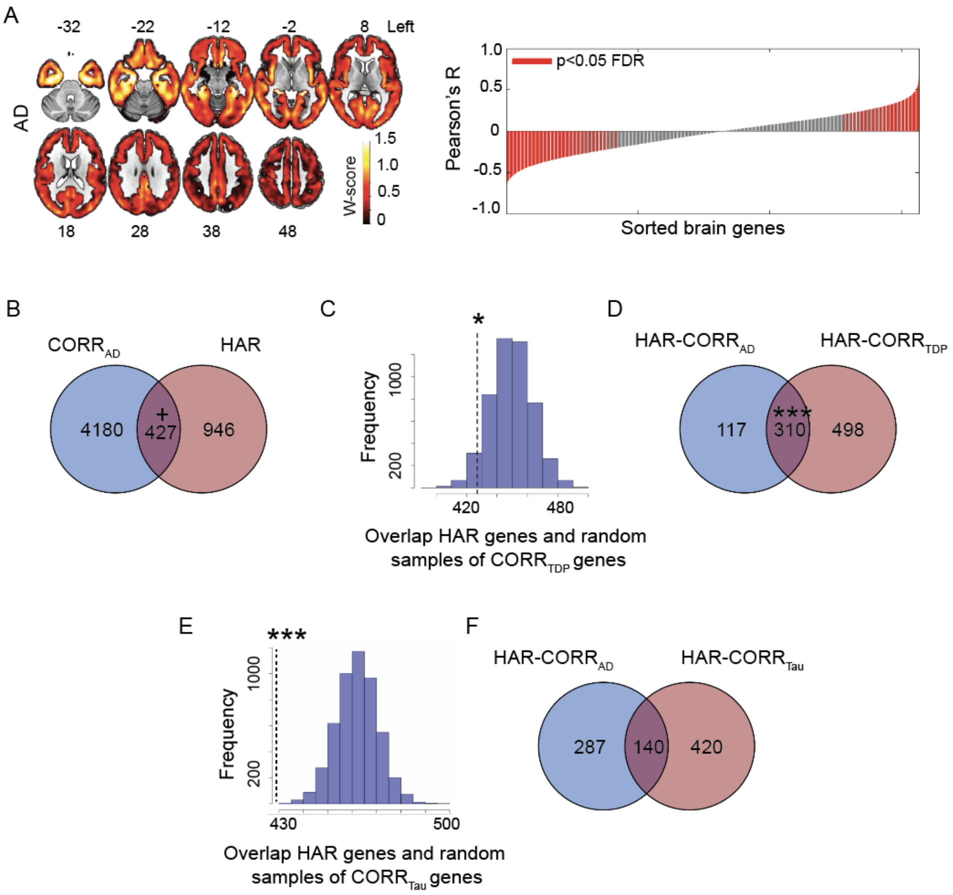

Supplementary Figure S8. Overlap of HAR genes and genes correlating with atrophy in Alzheimer's disease. (A) On the left, group-mean W-score map reflecting cortical atrophy in a sample of 147 amyloid- and tau-confirmed patients with Alzheimer's disease-type dementia (AD) from the Alzheimer's Disease Neuroimaging Initiative (ADNI). Warmer colors reflect greater cortical gray matter atrophy. The publicly available map (https://neurovault.org/collections/9297/) was resliced to the same dimensions as the Brainnetome atlas and W-scores of voxels located in the same Brainnetome parcel were averaged. Bar plot on the right shows the Pearson correlation coefficients between the regional gene expression and regional atrophy in AD; red bars indicate significantly correlated genes, FDR adjusted p <0.05. This approach identified 4,607 genes significantly correlating with atrophy in AD (2,943 negatively, 1,664 positively). **(B)** Of the 4,607 genes correlating with atrophy in Alzheimer's disease, 427 overlapped with HAR genes. This overlap did not reach significance (p = 0.082). **(C)** To control for atrophy-correlated gene list length, the overlap between HAR genes and randomly sampled lists of 4,607 genes correlating with atrophy in FTLD-TDP was calculated 5,000 times, resulting in a distribution of overlaps

between HAR and FTLD-TDP atrophy-correlated genes. We next counted how many overlaps were equal to or smaller than the overlap in AD (427) and divided this number by the 5,000 permutations to derive a *p* value of significance. The overlap between HAR genes and randomly sampled lists of 4,607 genes correlating with atrophy in FTLD-TDP was higher than the overlap between HAR genes and genes correlating with atrophy in Alzheimer's disease ($p = 0.052$). **(D)** Significant overlap between HAR genes correlating with atrophy in Alzheimer's disease and HAR genes correlating with atrophy in FTDL-TDP ($p < 0.005$). This analysis was performed by using the totality of HAR genes as background instead of the whole AHBA dataset of brain-expressed genes. **(E)** The overlap between HAR genes and randomly sampled list of 4,607 genes correlating with atrophy in FTLD-tau was calculated 5,000 times, resulting in a distribution of overlaps for both gene lists. We next counted how many overlaps were equal to or smaller than 427 and divided this number by the 5,000 permutations to derive a *p* value of significance. The overlap between HAR genes and randomly sampled lists of 4,607 genes correlating with atrophy in FTLD-tau was significantly higher than the overlap between HAR genes and genes correlating with atrophy in Alzheimer's disease ($p < 0.005$). **(F)** Nonsignificant overlap between HAR genes correlating with atrophy in Alzheimer's disease and HAR genes correlating with atrophy in FTLD-tau ($p = 1.00$). This analysis was performed by using the totality of brain-expressed HAR genes as background instead of the whole AHBA dataset of brain-expressed genes. $^{+}p = 0.082$ $^{*}p = 0.052$; *** $p < 0.005$.

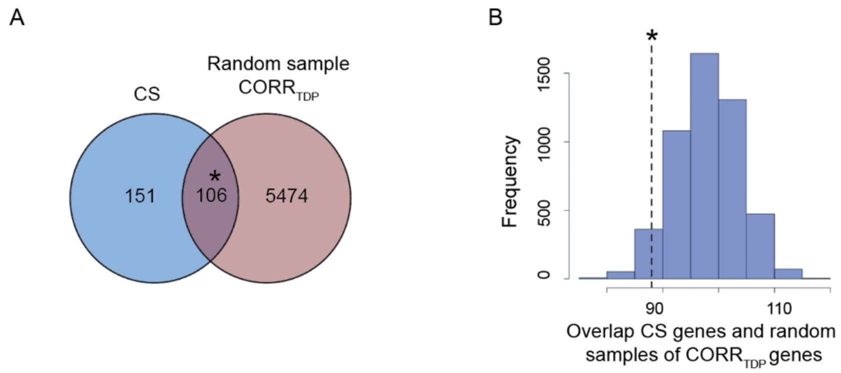

Supplementary Figure S9. Overlap between CS genes and random subsamples of genes correlating with atrophy in FTLD-TDP. (A) Example of a significant overlap of 106 genes shared between CS genes and a randomly sampled list of 5,580 genes correlating with atrophy in FTLD-TDP. **(B)** The overlap between CS genes and randomly sampled list of 5,580 genes correlating with atrophy in FTLD-TDP was calculated 5,000 times, resulting in a distribution of overlaps for both gene lists. We next counted how many overlaps were equal to or smaller than 88 and divided this number by the 5,000 permutations to derive a *p* value of significance. The overlap between CS genes and randomly sampled list of 5,580 genes correlating with atrophy in FTLD-TDP was significantly higher than the overlap between CS genes and 5,580 genes correlating with atrophy in FTLD-tau (n=88; dotted line). *$p < 0.05$.

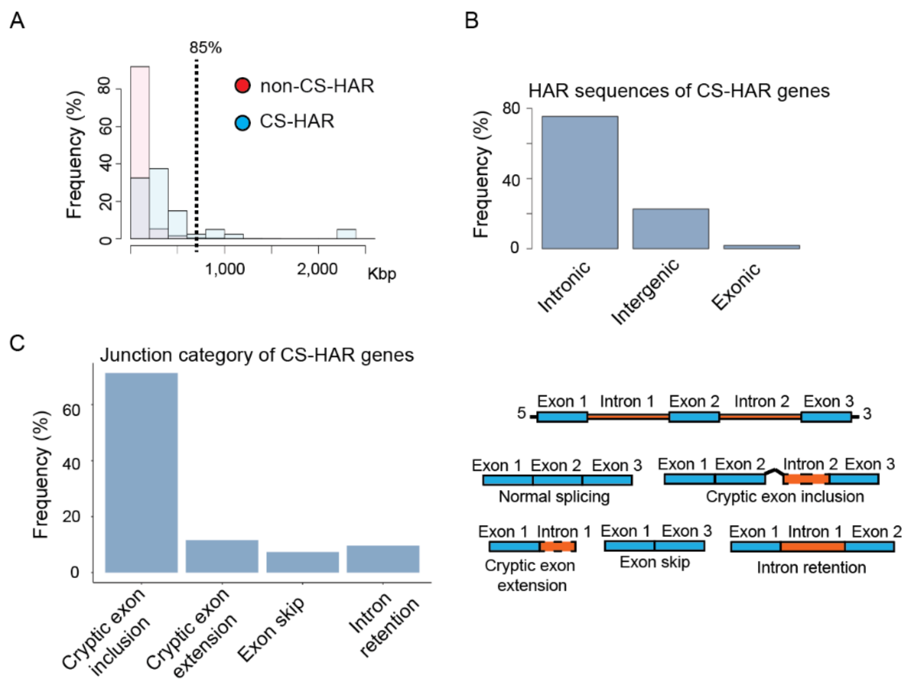

Supplementary Figure S10. CS-HAR genes are longer than other brain expressed genes, contain mostly intronic HAR sequences, and cryptic exon cassettes are the most common mis-splicing event. (A) CS-HAR genes tended to be longer than other genes in the genome, although 85% of CS-HAR genes displayed lengths comparable to those of non-CS-HAR genes. **(B)** The majority of HAR sequences of CS-HAR genes were intronic, followed by intergenic, with only a small fraction being exonic. **(C)** Junction category of miss-splicing events for genes derived from iPSC studies. The panel on the right provides a schematic overview of the distinct splicing events.

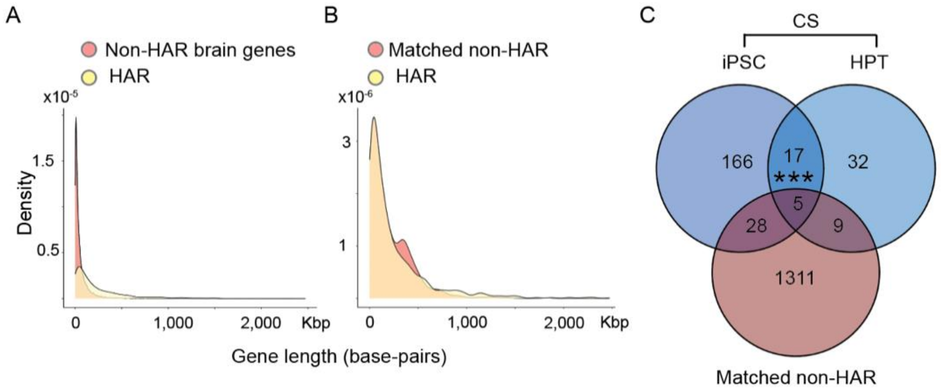

Supplementary Figure S11. Overlap of CS genes and length-matched non-HAR genes. (A) Length distributions of HAR brain genes and non-HAR brain genes. **(B)** Length distributions of HAR brain genes and length-matched non-HAR brain genes. **(C)** Overlap between length-matched non-HAR brain genes and CS genes. ***$p < 0.0005$

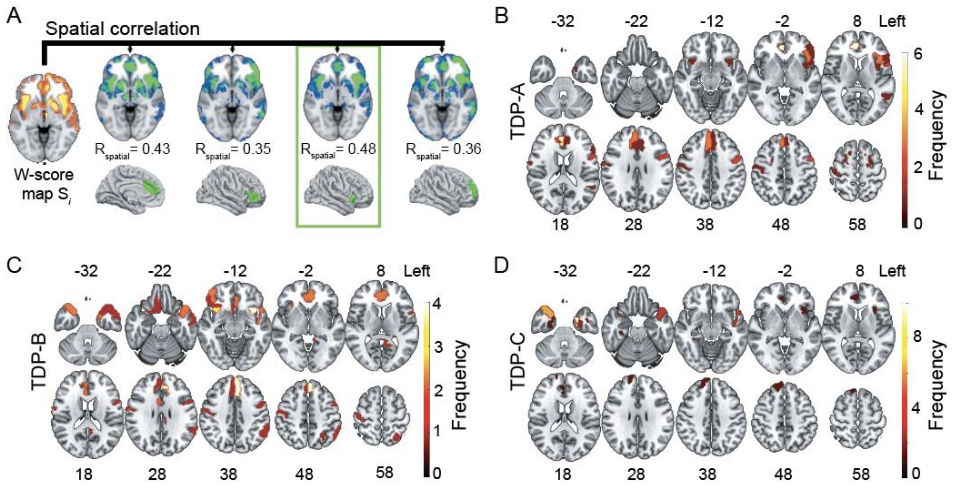

Supplementary Figure S12. Epicenter detection and neuroanatomical distribution in FTLD-TDP subtypes. (A) We correlated individual atrophy W-score maps with a library of functional connectivity maps derived in healthy older adults to identify epicenters in FTLD-TDP. The seed used to generate the functional connectivity map with the highest spatial similarity to the individual atrophy map was considered the epicenter for that patient. Frequency maps reflecting the spatial distribution of epicenters across FTLD-TDP subtypes. The most common disease epicenter for patients with FTLD-TDP-A was in the right anterior cingulate cortex **(B)**; for FTLD-TDP-B in the left anterior insula **(C)**; and for FTLD-TDP-C in the left inferior temporal lobe **(D)**. Panel A adapted with permission from[53].

Supplementary Appendix

Abagen Code:

$ abagen --output-file Brainnetome_237ROIs_expression.csv Brainnetome_237ROIs.nii.gz

Abagen Report:

Regional microarry expression data were obtained from 6 post-mortem brains (1 female, ages 24.0--57.0, 42.50 +/- 13.38) provided by the Allen Human Brain Atlas (AHBA, https://human.brain-map.org; [H2012N]). Data were processed with the abagen toolbox (version 0.1.3; https://github.com/rmarkello/abagen) using a 273-region volumetric atlas in MNI space.

First, microarray probes were reannotated using data provided by [A2019N]; probes not matched to a valid Entrez ID were discarded. Next, probes were filtered based on their expression intensity relative to background noise [Q2002N], such that probes with intensity less than the background in >=50.00% of samples across donors were discarded. When multiple probes indexed the expression of the same gene, we selected and used the probe with the most consistent pattern of regional variation across donors (i.e., differential stability; [H2015N]), calculated with:

$$\Delta S(p) = \frac{1}{\binom{N}{2}} \sum_{i=1}^{N-1} \sum_{j=i+1}^{N} \rho[B_i(p),]B_j(p)$$

Where ρ is Spearman's rank correlation of the expression of a single probe, p, across regions in two donors B_i and B_j and N is the total number of donors. Here, regions correspond to the structural designations provided in the ontology from the AHBA.

The MNI coordinates of tissue samples were updated to those generated via non-linear registration using the Advanced Normalization Tools (ANTs; https://github.com/chrisfilo/alleninf). Samples were assigned to brain regions in the provided atlas if their MNI coordinates were within 2 mm of a given parcel. All tissue samples not assigned to a brain region in the provided atlas were discarded.

Inter-subject variation was addressed by normalizing tissue sample expression values across genes using a robust sigmoid function [F2013J]:

$$x_{norm} = \frac{1}{1 + \exp\left(-\frac{x - \langle x \rangle}{IQR_z}\right)}$$

where $\langle x \rangle$ is the median and IQR_z is the normalized interquartile range of the expression of a single tissue sample across genes. Normalized expression values were then rescaled to the unit interval:

$$x_{scaled} = \frac{x_{norm} - min(x_{norm})}{max(x_{norm}) - min(x_{norm})}$$

Gene expression values were then normalized across tissue samples using an identical procedure. Samples assigned to the same brain region were averaged separately for each donor and then across donors, yielding a regional expression matrix.

REFERENCES:

[A2019N]: Arnatkevičiūtė, A., Fulcher, B. D., & Fornito, A. (2019). A practical guide to linking brain-wide gene expression and neuroimaging data. Neuroimage, 189, 353-367.

[F2013J]: Fulcher, B. D., Little, M. A., & Jones, N. S. (2013). Highly comparative time-series analysis: the empirical structure of time series and their methods. Journal of the Royal Society Interface, 10(83), 20130048.

[H2012N]: Hawrylycz, M. J., Lein, E. S., Guillozet-Bongaarts, A. L., Shen, E. H., Ng, L., Miller, J. A., ... & Jones, A. R. (2012). An anatomically comprehensive atlas of the adult human brain transcriptome. Nature, 489(7416), 391-399.

[H2015N]: Hawrylycz, M., Miller, J. A., Menon, V., Feng, D., Dolbeare, T., Guillozet-Bongaarts, A. L., ... & Lein, E. (2015). Canonical genetic signatures of the adult human brain. Nature Neuroscience, 18(12), 1832.

[Q2002N]: Quackenbush, J. (2002). Microarray data normalization and transformation. Nature Genetics, 32(4), 496-501.
